# Strategies for Stability Enhancement of Metal Halide Perovskite Nanocrystals for Efficient and Stable Perovskite LEDs

**DOI:** 10.3390/ma19214497

**Published:** 2026-10-26

**Authors:** Kyoung-Been Shin, Ji-Eun Kim, Gi-Yeon Park, Seo-Yeon Choi, Sol Choi, Min-Ho Park

**Affiliations:** 1 Department of Materials Science and Engineering, Soongsil University, 369 Sangdo-ro, Dongjak-gu, Seoul 06978, Republic of Korea; 2 Department of Convergence of Energy Policy and Technology, Soongsil University, 369 Sangdo-ro, Dongjak-gu, Seoul 06978, Republic of Korea

**Keywords:** perovskite light-emitting diodes, stability, ligand engineering, encapsulation, core–shell, crosslinking

## Abstract

Metal halide perovskite nanocrystals (PeNCs) exhibit narrow emission spectral linewidths, high color purity, and low-temperature solution processability, making them promising emitters for next-generation light-emitting diodes (LEDs). However, their practical application remains limited by the ionic nature of the PeNC surface, which leads to ligand desorption, defect formation, and structural degradation under ambient air exposure and device operation. This review focuses on three material design strategies of ligand engineering, encapsulation, and crosslinking to overcome this intrinsic instability of PeNCs. Ligand engineering employs aliphatic ionic, metal-coordinated Z-type, and aromatic conjugated ligands to strengthen the surface binding and passivate point defects at the molecular level. Encapsulation provides physical barriers against moisture, oxygen, and thermal stress using inorganic shells (SiO_2_, ZrO_2_, ZnS, and CdS) or polymer matrices. Crosslinking chemically links adjacent nanocrystals or ligand chains, forming insoluble networks that reduce ligand desorption, solvent damage, and ion migration. We reviewed how these strategies affect the photoluminescence (PL) retention and colloidal stability of PeNCs, charge transport, and operational stability in perovskite light-emitting diodes (PeLEDs). Based on this review, we summarize the material design principles for developing highly stable PeNC-based LEDs.

## 1. Introduction

Metal halide perovskite nanocrystals (PeNCs) with the formula ABX_3_ have emerged as promising candidates for next-generation light-emitting diodes (LEDs) owing to their narrow full width at half maximum (FWHM) of the emission spectrum, high photoluminescence quantum yield (PLQY), high color purity, defect-tolerant electronic structure, and solution processability at low temperatures [1,2,3,4,5,6,7,8]. However, the inherent instability of PeNCs remains the primary obstacle to their practical application in perovskite light-emitting diodes (PeLEDs) [9,10]. This instability originates from multiple, often coupled factors. The soft and ionic nature of the lattice makes PeNCs prone to ligand desorption and ion migration, while their high surface-to-volume ratio increases their susceptibility to defect formation under moisture, oxygen, heat, and electrical stress [11,12,13].

Defects, such as lead and halide vacancies, primarily cause performance degradation in PeLEDs [7,9,10]. These vacancies create trap sites within the bandgap that decrease the PLQY, broaden the FWHM of the emission spectrum, and trigger the lattice decomposition of PeNCs. Moreover, vacancies at the grain boundaries accelerate ion migration, causing efficiency loss and device degradation [6,7,14]. As a result, PeLEDs typically exhibit rapid luminance decay, with operational lifetimes often limited to hours or less.

**Figure 1:**
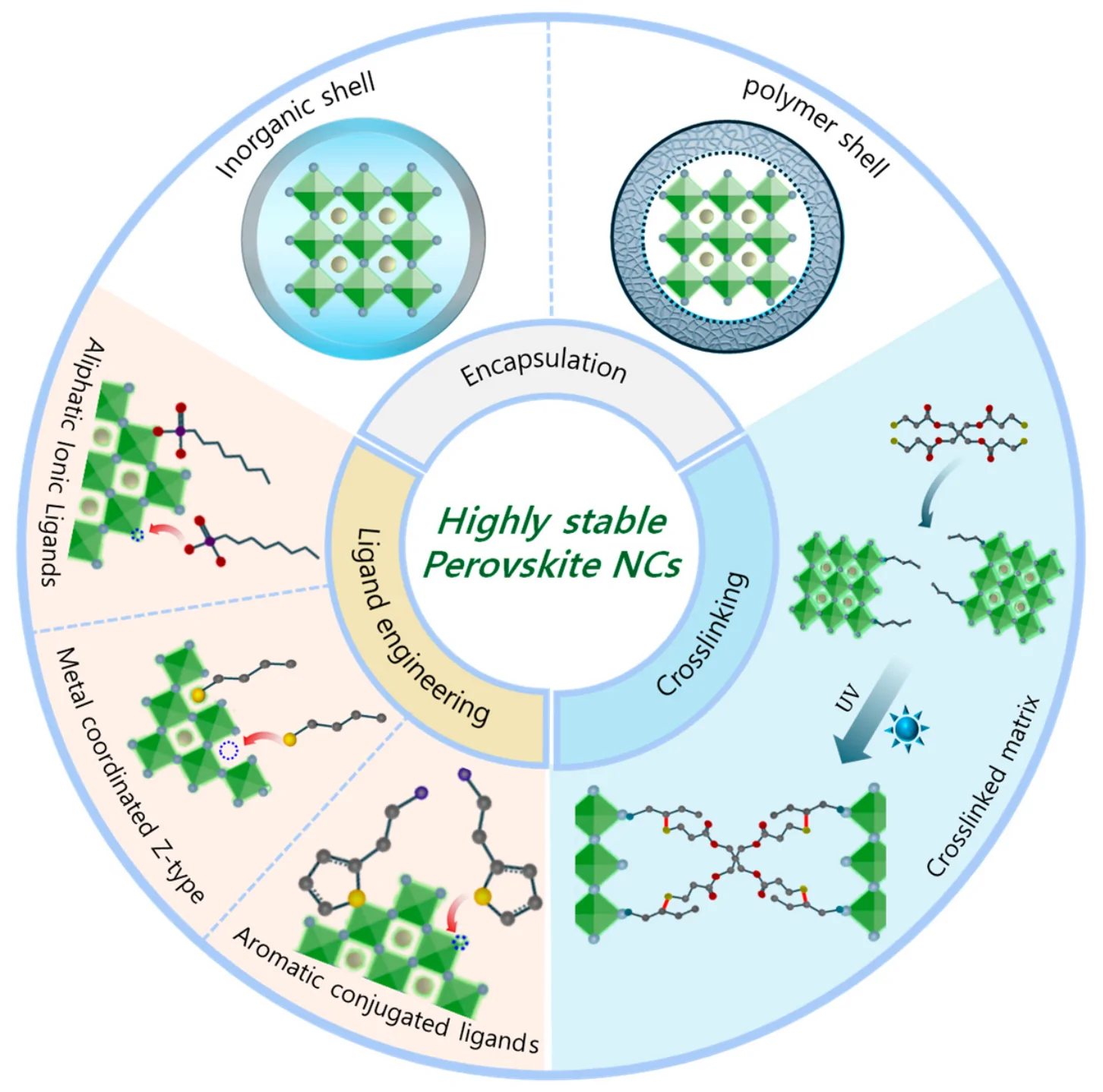
Illustration of three strategies for PeNC stabilization.

To address these challenges, various strategies of ligand engineering, encapsulation, and crosslinking have been reported for PeNC stabilization (Figure 1). In this review, we examine these three strategies using representative examples. For each strategy, we discuss how it improves the environmental and colloidal stability of PeNCs and how these improvements affect their optical properties, carrier dynamics, and operational lifetimes of PeLEDs. We then present PeNC design guidelines for efficient and stable PeLEDs.

## 2. Ligand Engineering

Ligands are molecules that bind to the surface of NCs, forming a capping layer around each particle [15,16,17]. They can regulate precursor reactivity and control nucleation and crystal growth during PeNC synthesis. They also prevent particle aggregation while providing excellent colloidal stability and solution dispersibility, thereby enabling high PLQY and uniform film formation of the PeNCs.

Oleic acid (OA) and oleylamine (OAm) are the most widely used surface ligands for PeNCs. They undergo an acid–base reaction to form oleate^−^ and oleylammonium^+^, which bind to the ionic PeNC surface. While oleate^−^ passivates the cationic sites and oleylammonium^+^ passivates the halide-related sites, their long C18 alkyl chains further provide a steric barrier that suppresses aggregation and maintains the colloidal stability of PeNCs [18,19]. However, the conventional OA/OAm ligand system suffers from several limitations. First, the ligands can easily detach from the PeNC surface during purification, ligand exchange, and storage processes because their surface binding is driven by a dynamic adsorption–desorption equilibrium. Second, the long hydrocarbon chains are electrically insulating, weakening the electronic coupling between neighboring PeNCs, thereby hindering charge transport. To overcome these intrinsic limitations, several ligand engineering strategies incorporating aliphatic, metal-coordinated Z-type, and aromatic ligands have been developed. These strategies focus on achieving stronger surface binding and more effective passivation of surface defects than those of OA/OAm ligands. Ligand-engineered PeNCs facilitate charge transport and suppress ion migration, thereby improving the performance and stability of both PeNCs and PeLEDs.

### 2.1. Aliphatic Ionic Ligands

Aliphatic ionic ligands, including phosphonic acid-based, quaternary ammonium, diammonium, and zwitterionic molecules, feature flexible carbon chains with charged head groups that tightly bind to the PeNC lattice. These ionic species effectively suppress ligand desorption and surface defect formation, enhancing the environmental stability of the PeNC film and facilitating charge transport in PeLED applications.

Phosphonic acid-based ligands have attracted attention because of their strong binding affinity for surface Pb^2+^, which effectively suppresses the desorption of the ligand. In 2018, Tan et al. demonstrated that octylphosphonic acid (OPA) significantly improved the stability of CsPbBr_3_ NCs compared to the conventional OA/OAm ligand systems [20]. Thermogravimetric analysis (TGA) showed that OPA-capped PeNCs exhibited significantly lower weight losses than OA/OAm-capped PeNCs, retaining over 95% of their initial weight at 450 °C compared to only 70% for OA/OAm-capped PeNCs. This indicates a stronger ligand binding and higher ligand retention. OPA-capped PeNCs also maintained a much higher PLQY than the OA/OAm-capped PeNCs after repeated purification cycles. After four washing cycles, the OPA-capped PeNCs retained >80% PLQY, whereas only 20% of the OA/OAm-capped PeNCs retained their PLQY. Even after eight washing cycles, the OPA-capped PeNCs maintained a 70% PLQY. This superior performance is consistent with the high binding affinity of the phosphonic acid head group, which suppresses ligand desorption during solvent washing, thereby reducing the surface degradation. OPA-capped PeNC films also retained most of their initial PL intensity during prolonged ambient storage, whereas OA/OAm-capped PeNC films showed a rapid decrease in PL intensity over time. This indicates that OPA effectively suppresses surface degradation and significantly enhances the long-term optical stability of PeNCs.

Around the same time, Park et al. reported that quaternary ammonium bromide (QAB) ligands compensate for surface halide vacancies and form shorter ligand shells than OA/OAm ligands, thereby improving the electronic coupling between neighboring NCs and reducing charge-transporting resistance [21]. CsPbBr_3_ NCs capped with dioctyldimethylammonium bromide (DOAB) showed an increase in PLQY from 38% to 70% compared to OAm-capped PeNCs and retained approximately 70% of their initial PLQY after 6 days under ambient conditions. Those with didecyldimethylammonium bromide (DDeAB), which has a longer carbon chain than DOAB, achieved a maximum external quantum efficiency (EQE) of 9.71%, corresponding to a 16-fold enhancement in EQE compared to OAm-capped CsPbBr_3_ NC-based PeLEDs.

**Figure 2:**
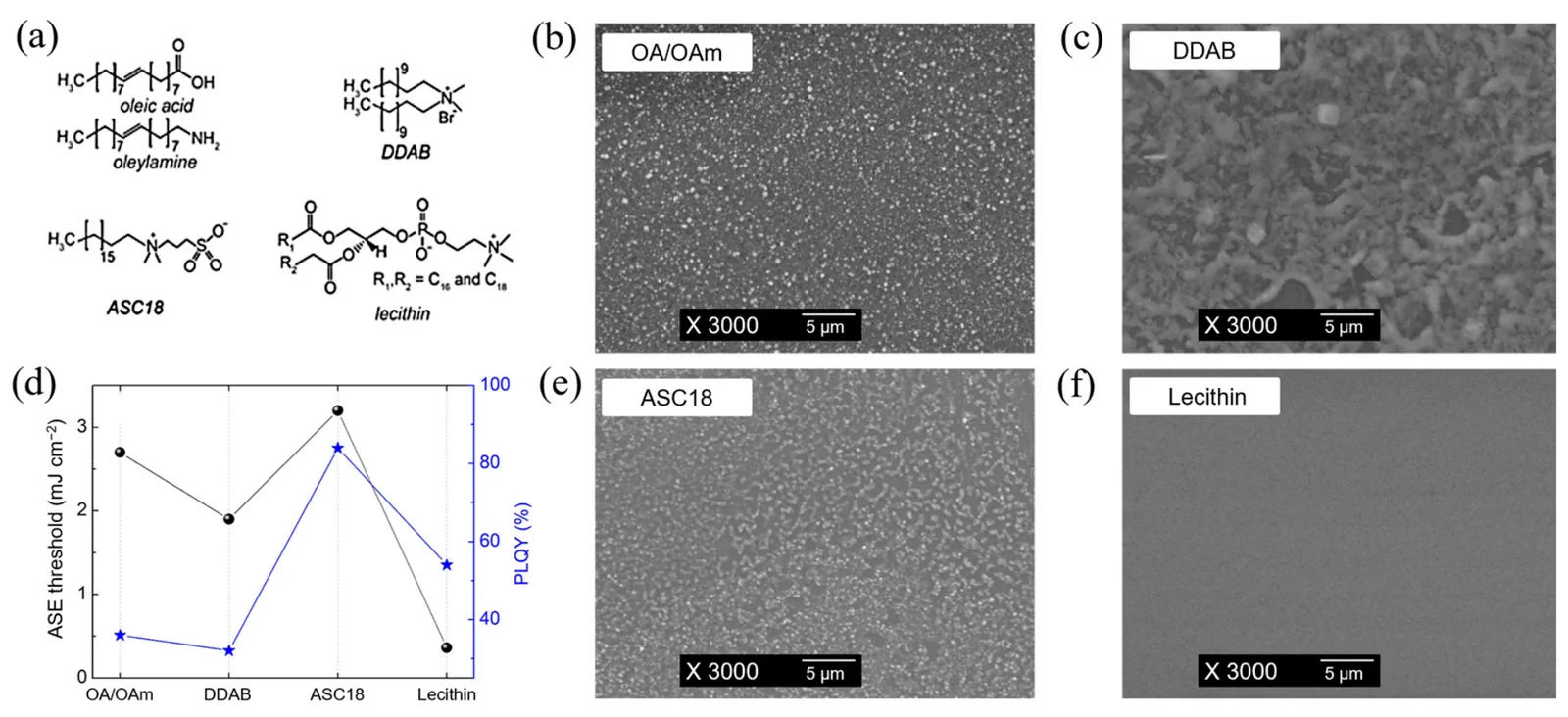
(**a**) Chemical structures of different capping ligands. Scanning electron microscope (SEM) images of the PeNC films for (**b**) OA/OAm, (**c**) didodecyldimethylammonium bromide (DDAB), (**e**) 3-(N,N-dimethyloctadecylammonio)-propanesulfonate (ASC18), and (**f**) Lecithin. (**d**) Amplified spontaneous emission (ASE) threshold (black circles) and PLQY (blue stars) of PeNC films. Reprinted from Ref. [22]; copyright ACS Applied Nano Materials 2025.

Recently, Milanese et al. evaluated the optical properties and stability of CsPbBr_3_ NC films passivated with different capping ligands (Figure 2a). Regarding the solid-state PLQY, multidentate ligands such as 3-(N,N-dimethyloctadecylammonio)-propanesulfonate (ASC18) and lecithin provided superior surface protection via the chelate effect and showed high PLQY, as indicated by the blue stars in Figure 2d. As the short-chain didodecyldimethylammonium bromide (DDAB) and OA/OAm offered lower PLQYs (32% and 36%, respectively), ASC18-capped PeNC films achieved the highest PLQY of 84%, followed by lecithin at 54%. Moreover, zwitterionic lecithin exhibited a smooth, defect-free film surface, indicating the most uniform film morphology (Figure 2b,c,e,f). Consequently, the lecithin-capped film achieved the lowest amplified spontaneous emission (ASE) threshold, as indicated by the black circles in Figure 2d.

**Figure 3:**
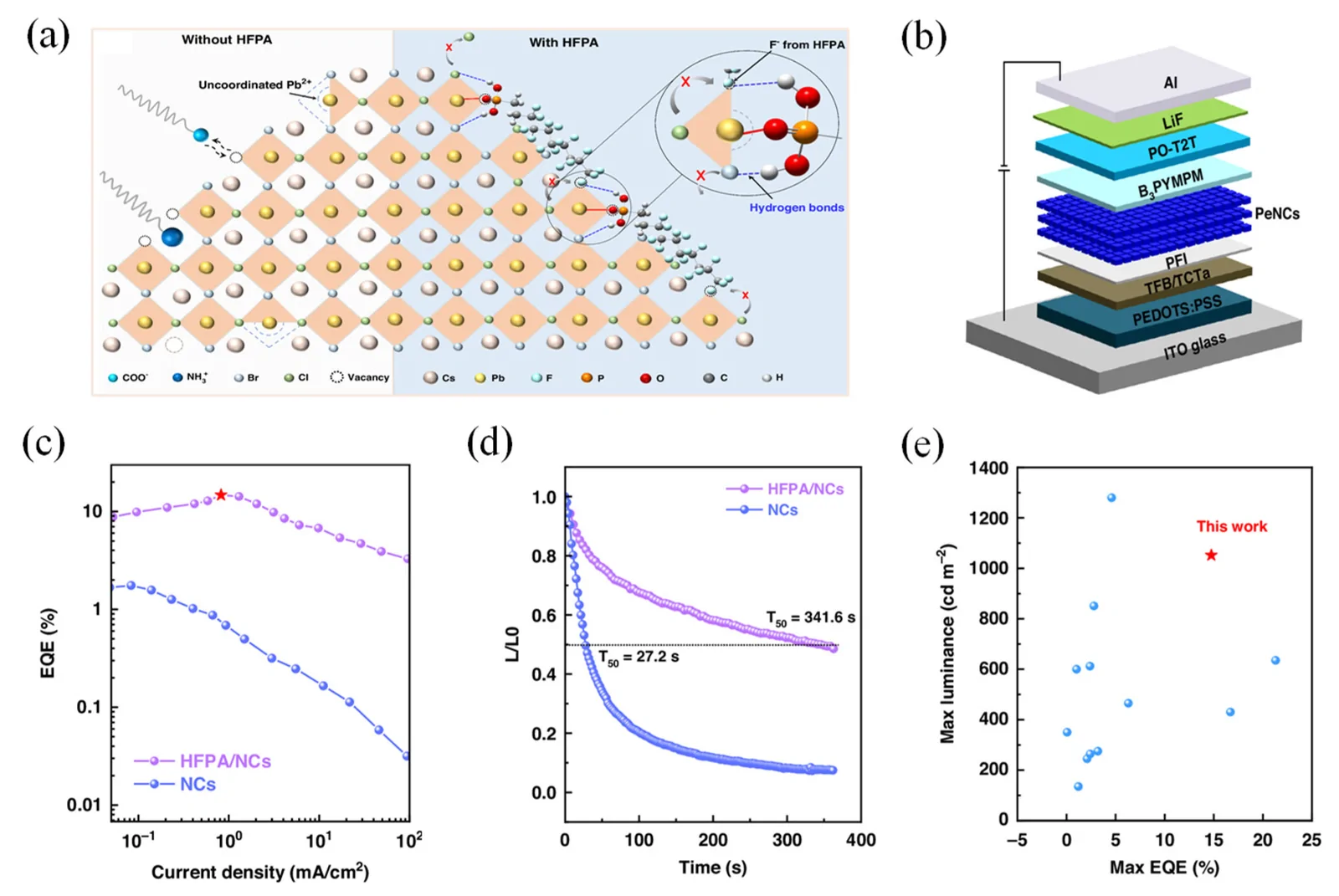
(**a**) Illustration of defect passivation mechanism of PeNCs and (**b**) PeLED structure. (**c**) EQE-current density curves, (**d**) operational lifetimes, (**e**) maximum luminance and EQE of PeLEDs. Reprinted from Ref. [23]; copyright Light: Science & Applications 2026.

The incorporation of highly electronegative elements and alternative anchoring groups has emerged as an effective approach for enhancing PeLED stability. Maimaitizi et al. demonstrated that fluorinated short-chain ligands can comprehensively suppress defect formation and ion migration in mixed halide PeNCs. The use of (1H,1H,2H,2H-heptadecafluorodec-1-yl)phosphonic acid (HFPA) during the hot-injection synthesis process allows the molecule to bind to the CsPb(Br/Cl)_3_ NC surface through a multi-site interaction network. The phosphonic acid head group coordinates with under-coordinated surface Pb^2+^ sites to eliminate cationic trap states, while the adjacent hydroxyl (-OH) segments form hydrogen bonds with the surface halides to suppress vacancy formation (Figure 3a). Simultaneously, the highly electronegative fluorine atoms in the molecular tail induce local lattice rigidification and improve the stability of the octahedral framework, establishing effective defense against interstitial ion transport. Because the fluorinated HFPA ligand maintains a relatively short chain length compared to OA/OAm, sufficient electronic coupling between adjacent PeNCs is preserved, which lowers the charge-hopping barrier and facilitates efficient carrier injection into the PeNCs. Consequently, the optimized pure-blue PeLEDs emitting at 467 nm achieved a peak EQE of 14.8% and a maximum luminance of 1052 cd/m^2^, representing a respective 9-fold and 10-fold enhancement over the pristine PeLEDs (Figure 3b,c,e). Furthermore, the effective suppression of ion migration prolonged the operational half-lifetime (T_50_) from 27.2 s to 341.6 s, successfully combining trap-state mitigation with lattice reinforcement to improve both the electroluminescence (EL) efficiency and operational stability of the PeLED (Figure 3d).

Overall, advanced aliphatic ionic ligands, including phosphonic acids, quaternary ammonium salts, zwitterions, and fluorinated short-chain groups, address the key challenges of defect formation and operational stability in PeNCs and PeLED applications. Strategic modification of the head-group coordination and chain length enables simultaneous surface passivation, reduces charge-transfer resistance, and suppresses ion migration under bias. Balancing the high chemical affinity with efficient carrier injection provides a robust framework for developing environmentally resilient and durable PeLEDs.

However, ligand engineering may also be accompanied by side effects. Studies of PeLEDs using DDAB-treated PeNCs have reported that excessive ligand density can produce an insulating layer that impedes charge injection into the PeNC layer in PeLEDs [24]. Conversely, excessive removal of DDAB ligands during washing process can generate additional surface defects that can act as new trap states and reduce the stability of PeNCs [25]. Therefore, ligand engineering should include careful control of ligand density and processing conditions to balance surface passivation in PeNCs with charge injection and transport in PeLEDs.

### 2.2. Metal-Coordinated Ligands

Metal-coordinating ligands have emerged as an effective strategy for further improving the surface chemistry of PeNCs. Lewis-acidic metal centers in metal-coordinating ligands can directly coordinate with undercoordinated Pb^2+^ sites and halide vacancies, thereby enabling more efficient defect passivation and surface coordination. Representative approaches include Lewis acid-based Z-type ligands, which have demonstrated significant improvements in defect passivation, photoluminescence (PL) efficiency, carrier lifetime, and long-term stability.

**Figure 4:**
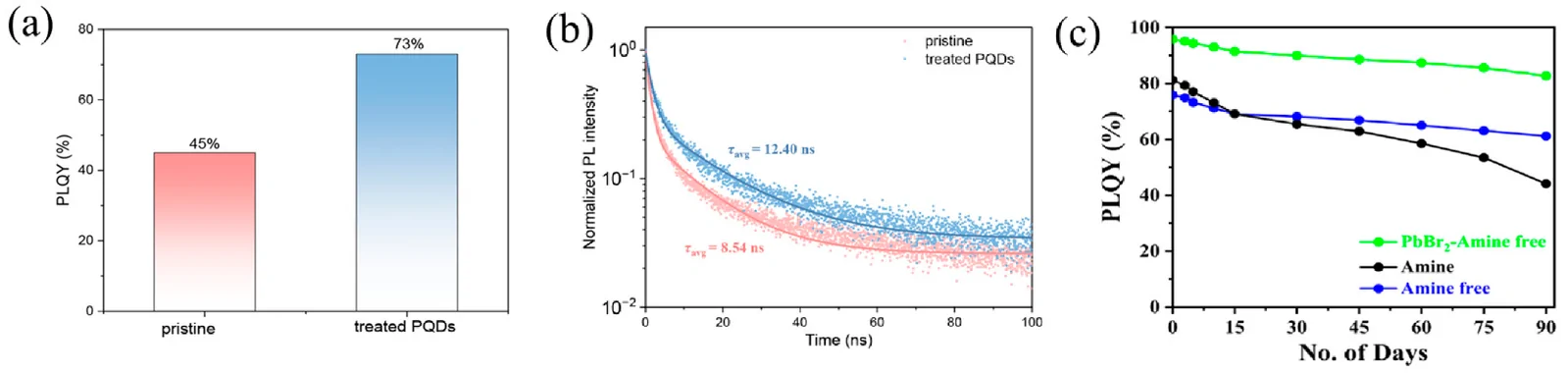
(**a**) PLQY of PeNCs before and after Zn-octanoate treatment. (**b**) Time-resolved photoluminescence (TRPL) decay curves showing a prolonged carrier lifetime after Z-type ligand passivation. Reprinted from Ref. [27]; copyright ACS Applied Materials & Interfaces 2024. (**c**) Long-term storage stability (PLQY retention over 90 days) of PbBr_2_-treated CsPbBr_3_ NCs compared with untreated samples. Reprinted from Ref. [28]; copyright Journal of Physical Chemistry C 2022.

Ligands are classified as L-type (Lewis bases that donate electron pairs), X-type (anionic surface-binding species), or Z-type (Lewis acids that accept electrons). Among them, Z-type ligands coordinate strongly with surface Br^−^ ions, suppress excessive amine enrichment, and thereby stabilize the acid–base equilibrium at the PeNC surface more effectively than OA/OAm [26]. This regulation of surface chemistry also suppresses the formation of low-dimensional byproducts such as 2D nanoplatelets and promotes uniform PeNC growth, simultaneously improving optical properties and structural stability. Chen et al. reported deep-blue CsPbBr_3_ quantum dots (QDs) employing zinc octanoate. Instead of acting as a Zn^2+^ source, zinc octanoate, along with octanoate ligands, regulates the surface acid–base equilibrium and maintains the OA/OAm binding. Zn^2+^ coordinates strongly with surface Br^−^ species as a Lewis acid passivation center while suppressing excessive amine enrichment. This regulation inhibits the formation of low-dimensional nanoplatelets induced by amine accumulation and promotes more homogeneous deep-blue CsPbBr_3_ QD growth, thereby reducing the defect density and improving structural stability. Zn-octanoate treatment increased the PLQY of these QDs from 45% to 73% and extended the average carrier lifetime from 8.54 to 12.40 ns, consistent with the reduced surface trap density and suppressed non-radiative recombination (Figure 4a,b). The deep-blue emission characteristics were preserved despite the longer carrier lifetime, indicating that the improvement originated from surface defect passivation rather than changes in the underlying crystal structure.

Z-type passivation is not limited to Zn-based systems. Akhil et al. demonstrated that PbBr_2_ post-treatment effectively suppresses surface defects in amine-free CsPbBr_3_ NCs. PbBr_2_ provides Lewis-acid Pb^2+^ centers and serves as an additional Br^−^ source that passivates Br^−^ vacancies and stabilizes under-coordinated Pb^2+^ sites. The Br^−^-rich surface suppresses the regeneration of halide vacancies and induces defect healing while preserving the original amine-free surface of PeNCs. The PbBr_2_-treated samples retained 86.3% of their initial PLQY after 90 days of ambient storage, with the PLQY decreasing from 96% to 82% (Figure 4c). In comparison, untreated amine-free CsPbBr_3_ NCs showed a decrease from 75% to 61%, while amine-capped NCs exhibited the most pronounced degradation, decreasing from 81% to 46% over the same period. These results demonstrate that PbBr_2_ treatment not only increases the initial PLQY but also enables the NCs to maintain a substantially higher absolute PLQY during long-term storage.

Overall, metal-coordinating Z-type ligands enable precise surface defect passivation in PeNCs. By directly coordinating to undercoordinated halide and Pb^2+^ sites while regulating the surface acid–base equilibrium, these ligands suppress non-radiative recombination and prevent the formation of low-dimensional byproducts. The resulting improvements in the PLQY, carrier lifetime, and long-term storage stability demonstrate their utility in PeLED applications.

### 2.3. Aromatic Ligands

Aromatic ligands with short, conjugated backbones, often bearing electron-withdrawing substituents or existing in salt forms, bind to the PeNC surface and remain attached during anti-solvent washing. This interaction stabilizes the electrostatic potential of the PeNC films, which suppresses Pb^2+^ or halide vacancy formation and prevents phase transitions into non-perovskite structures. At the same time, the short conjugated chains reduce the charge-hopping barrier to facilitate efficient charge transport, leading to higher EQE and extended operational lifetime in PeLEDs.

Several studies have focused on thiophene derivatives for passivating defects at grain boundaries. Song et al. reported that post-treatment of CsPbI_3_ NC surface with 2-thiophenethylamine chloride (TEAC) induces surface reconstruction through cooperative interactions between molecular segments [29]. Although TEAC lacks a structural electron-withdrawing group, its protonated ammonium salt form induces a strong inductive electron-withdrawing effect, enabling the ligand to remain tightly bound to the PeNC surface even during rigorous anti-solvent washing. The primary amine cations occupy Pb^2+^ vacancies or coordinate with the Pb-halide octahedral framework, while chloride anions compensate for I^−^ vacancies generated during purification, and the sulfur lone pair of the thiophene ring forms a secondary Lewis acid–base bond with under-coordinated surface Pb ions. This dual-site anchoring suppresses electron trap states across multiple depths and mitigates non-radiative recombination. As a result, the passivation effect of TEAC increased the PL intensity relative to that of the control PeNC, and the TEAC-treated PeNC retained a high PLQY of 92.5% compared to 71.8% for the control after intensive purification, with longer carrier lifetimes of 68.37 ns compared to 57.48 ns for the control PeNC, as observed by time-resolved photoluminescence (TRPL). Moreover, surface defect passivation improved the charge injection and transport in PeLEDs, resulting in efficient radiative recombination of the charge carriers. The TEAC PeNC-based red PeLED achieved a high EQE of 17.3%, which was higher than that of the control PeLED with EQE of 12.3%, and a longer T_50_ of nearly 10 h without any EL shifts compared to 5 h of the control PeLED.

In addition to post-synthetic ligand exchange method, Pan et al. used density functional theory (DFT) calculations to examine the relationship between ligand steric configuration and surface affinity [30]. Because of its short-chain profile and dual anchoring sites (oxygen atoms of the –SO_2_NH_2_ group and hydrogen–halide bonding), thiophene-2-sulfonamide (2-ThSA) exhibited a higher binding energy (E_b_ = −2.57 eV) than the loosely bound native OA (−1.64 eV) and OAm (−2.02 eV) ligands. This stronger coupling raises the defect formation energy barrier of the I^−^ vacancy, thereby reinforcing the structural integrity of the CsPbI_3_ QDs. 2-ThSA-treated QDs showed a pure-red PL peak position that blue-shifted from 645 to 641 nm, correlating with a well-preserved quantum confinement effect from limited overgrowth during the hot-injection synthesis process. Additionally, the PLQY of the CsPbI_3_ QDs increased from 89.6% to near unity, which is supported by the TRPL decay transitioning from a bi-exponential profile with a lifetime of 10.4 ns to a single-exponential pathway with an increased lifetime of 15.1 ns, indicating suppressed trap-mediated non-radiative recombination. Femtosecond transient absorption (fs-TA) spectroscopy also showed a retarded exciton-bleaching recovery, suggesting that the photoexcited carriers successfully escaped the shallow traps to contribute to radiative recombination. As a result, the 2-ThSA-treated CsPbI_3_ QD exhibited improved ambient stability. While the PLQY of the pristine CsPbI_3_ QD solution rapidly dropped to only 8.9% within 20 days along with the formation of non-emissive yellow-phase precipitates, the 2-ThSA-treated CsPbI_3_ QD solution retained 85.5% of its initial PLQY even after 30 days.

**Figure 5:**
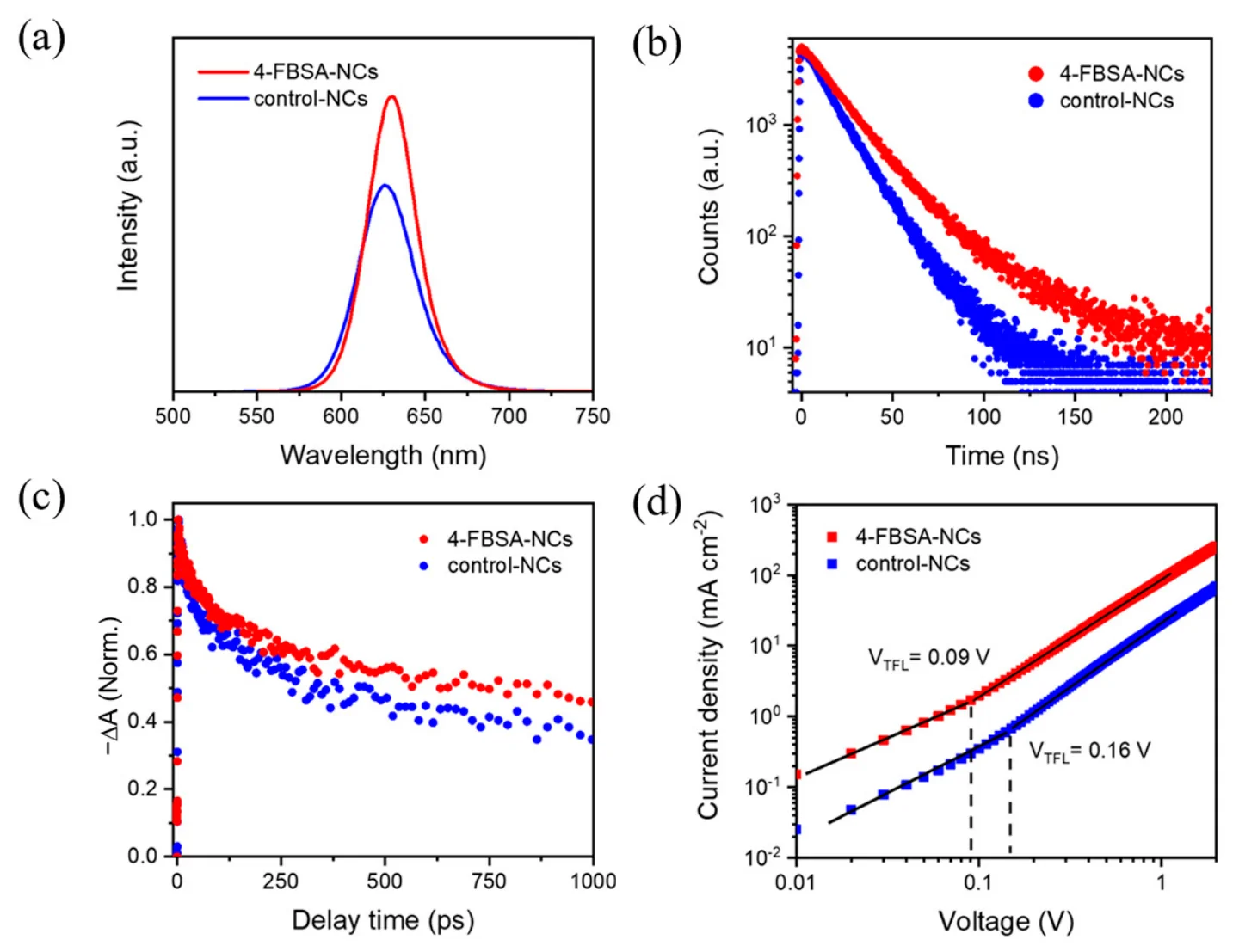
(**a**) PL spectra, (**b**) TRPL decay curves, (**c**) TA bleach recovery curves of PeNCs before and after treatment of 4-fluorobenzenesulfonamide (4-FBSA). (**d**) Current density–voltage curves of hole-only devices (HODs). Reprinted from Ref. [31]; copyright ACS Energy Letters 2024.

The structural vulnerability of red-emitting mixed-halide PeNCs, such as CsPb(Br/I)_3_ NCs, under continuous electrical bias remains an additional challenge, primarily driven by phase segregation and ion migration. For instance, unmodified mixed-halide PeLEDs typically show EL peak shifts from 626 to 648 nm at elevated current densities owing to halide displacement. To address this, Ye et al. designed a ligand that minimizes protonation under complex synthesis conditions. Surface treatment with 4-fluorobenzenesulfonamide (4-FBSA) forms a robust protective layer in which the electron-withdrawing fluorine atom and adjacent sulfonyl (–S(=O)_2_–) group reduce the Lewis basicity of the primary amine nitrogen, preventing proton interception by the surrounding organic acids. This proton-inert character allows 4-FBSA to act as a neutral capping ligand even under acid–amine coexisting conditions, forming a coordinate covalent bond with surface Pb^2+^, while the non-protonated amine segments form complementary hydrogen bonds with adjacent surface halides. Accordingly, the optical properties of the 4-FBSA-treated CsPb(Br/I)_3_ NCs were improved compared to those of the control CsPb(Br/I)_3_ NCs. The 4-FBSA CsPb(Br/I)_3_ NCs exhibited a pure-red emission peak at 630 nm, which was slightly red-shifted from 626 nm for the control CsPb(Br/I)_3_ NCs owing to the weakened quantum confinement induced by the larger diameter of 4-FBSA-treated CsPb(Br/I)_3_ NCs (Figure 5a). The FWHM decreased from 39 nm for the control CsPb(Br/I)_3_ NCs to 35 nm for the 4-FBSA-treated CsPb(Br/I)_3_ NCs, owing to a more uniform size distribution. Notably, the PLQY increased from 75% for control CsPb(Br/I)_3_ NCs to 93%. Furthermore, the TRPL curve showed a prolonged average PL lifetime of 22.5 ns for 4-FBSA-treated CsPb(Br/I)_3_ NCs compared to 16.8 ns for control CsPb(Br/I)_3_ NCs, indicating inhibited nonradiative recombination (Figure 5b). Consistently, the 4-FBSA-treated CsPb(Br/I)_3_ NCs exhibited slower ground-state bleaching decay dynamics within 1000 ps, indicating a more homogeneous energy distribution and fewer carrier traps (Figure 5c). Furthermore, hole-only device (HOD) evaluation revealed that the 4-FBSA-treated CsPb(Br/I)_3_ NCs exhibited higher hole current densities across various voltages than the control CsPb(Br/I)_3_ NCs, confirming more effective hole injection and reduced trap density within the film (Figure 5d). Driven by these improved film properties, the 4-FBSA-treated CsPb(Br/I)_3_ NC-based PeLEDs showed significantly improved overall performance, including an EQE of 21.8% from 11.3% for the control, and a maximum luminance of 21,590 cd/m^2^ from 6270 cd/m^−2^.

Overall, precisely designed aromatic fluorinated ligands provide a versatile route for simultaneously mitigating surface defects, suppressing ion migration, and preserving charge transport in PeNCs. The improved surface stability results in improved device efficiencies and extended operational lifetimes of the ligand-engineered PeLEDs.

## 3. Encapsulation Engineering

Encapsulation is an effective approach for improving the stability of PeNCs. Unlike ligand engineering, which directly targets atomic coordination at the PeNC surface, encapsulation engineering physically isolates the PeNC framework within an inorganic or polymeric matrix. This minimizes direct exposure to moisture, oxygen, heat, and other environmental factors while preserving the optical properties of the PeNCs. Consequently, degradation processes can be effectively suppressed, leading to enhanced long-term stability. Encapsulation strategies are generally divided into inorganic shell encapsulation and polymer encapsulation, which are discussed in the following sections.

### 3.1. Inorganic Shell

Among the reported inorganic encapsulation materials, SiO_2_ has been extensively studied owing to its excellent chemical stability, optical transparency, and moisture resistance [32]. There are SiO_2_ encapsulation strategies including solution-based core–shell synthesis and plasma-enhanced atomic layer deposition (PE-ALD).

**Figure 6:**
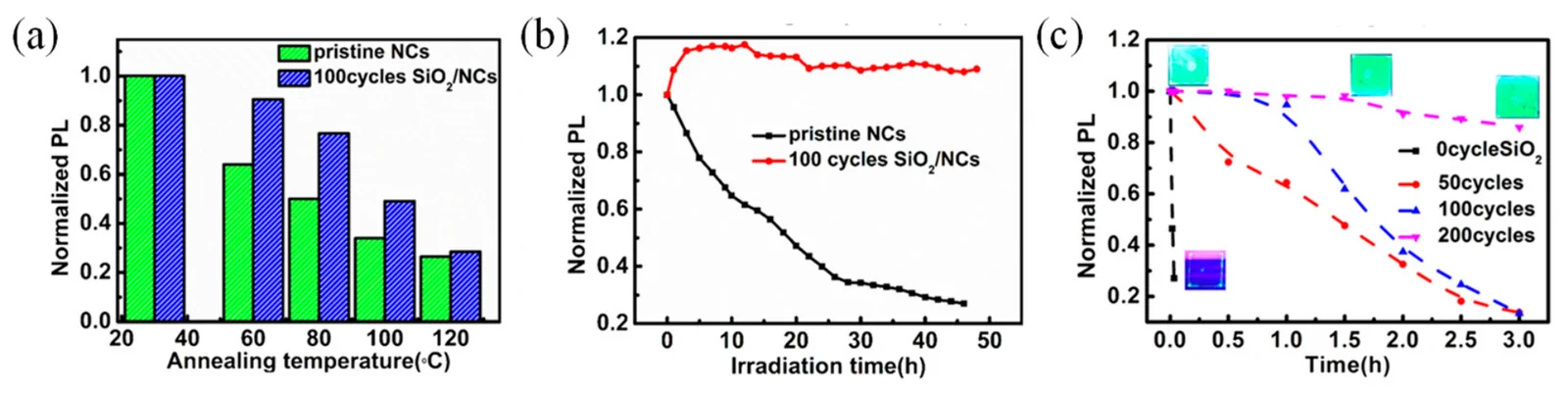
(**a**) Thermal stability, (**b**) photostability of pristine and SiO_2_-coated CsPbBr_3_ NC films via PE-ALD process. (**c**) water stability of CsPbBr_3_ NC films coated with different numbers of SiO_2_ ALD cycles, with photographs recorded under ultraviolet (UV) illumination. Reprinted from Ref. [34]; copyright ACS Applied Materials & Interfaces 2020.

In 2018, Zhong et al. synthesized core–shell-type CsPbBr_3_@SiO_2_ NCs using a one-pot method, in which SiO_2_ was formed in situ around ligand-capped CsPbBr_3_ NCs to generate a compact protective inorganic shell [33]. The resulting SiO_2_ shell effectively isolates the CsPbBr_3_ core from external moisture and oxygen while maintaining the optical properties of the CsPbBr_3_ NCs. The effectiveness of the SiO_2_ shell was reflected in the carrier recombination dynamics. The average PL lifetime of the SiO_2_-coated CsPbBr_3_ NCs increased from 12.4 ns for pristine CsPbBr_3_ NC to 16.8 ns, indicating that the surface trap states were effectively passivated and trap-assisted non-radiative recombination was suppressed [33]. An alternative SiO_2_ encapsulation strategy based on PE-ALD was reported by Jing et al. The low-temperature PE-ALD process enables the conformal deposition of a dense SiO_2_ layer on PeNC films without significantly affecting the surface ligands or PL properties. After SiO_2_ coating, by using 100 ALD cycles, the thermal stability improved at 60 ~ 120 °C compared to that of the pristine NC film (Figure 6a). The SiO_2_-coated CsPbBr_3_ NCs also maintained a nearly constant PL intensity after 48 h of continuous ultraviolet (UV) irradiation (~100 mW/cm^2^), whereas pristine CsPbBr_3_ NCs retained only 27% of their initial PL intensity because of ligand desorption and surface degradation (Figure 6b). Furthermore, the SiO_2_ shell served as an effective moisture barrier. While the pristine CsPbBr_3_ NCs decomposed within 60 s upon contact with water, the SiO_2_-coated CsPbBr_3_ NCs prepared with 200 ALD cycles maintained their PL emission even after 3 h of immersion in water (Figure 6c).

Another representative oxide shell is ZrO_2_, which also effectively improves the photophysical properties and environmental stability of PeNCs. Janus CsPbBr_3_/ZrO_2_ heterostructure PeNCs were synthesized by partially coating the surface of CsPbBr_3_ NCs with ZrO_2_, forming a type-I heterostructure that confines charge carriers within the CsPbBr_3_ core and effectively passivates surface defects [35]. The PLQY increased from 63% to 90%, and the average PL lifetime increased from 12.9 to 66.1 ns after ZrO_2_ coating. The ZrO_2_-coated CsPbBr_3_ NCs also exhibited enhanced resistance to polar solvent treatment compared to pristine CsPbBr_3_ NCs, indicating that partial ZrO_2_ encapsulation effectively improved the optical and environmental stabilities of PeNCs.

**Figure 7:**
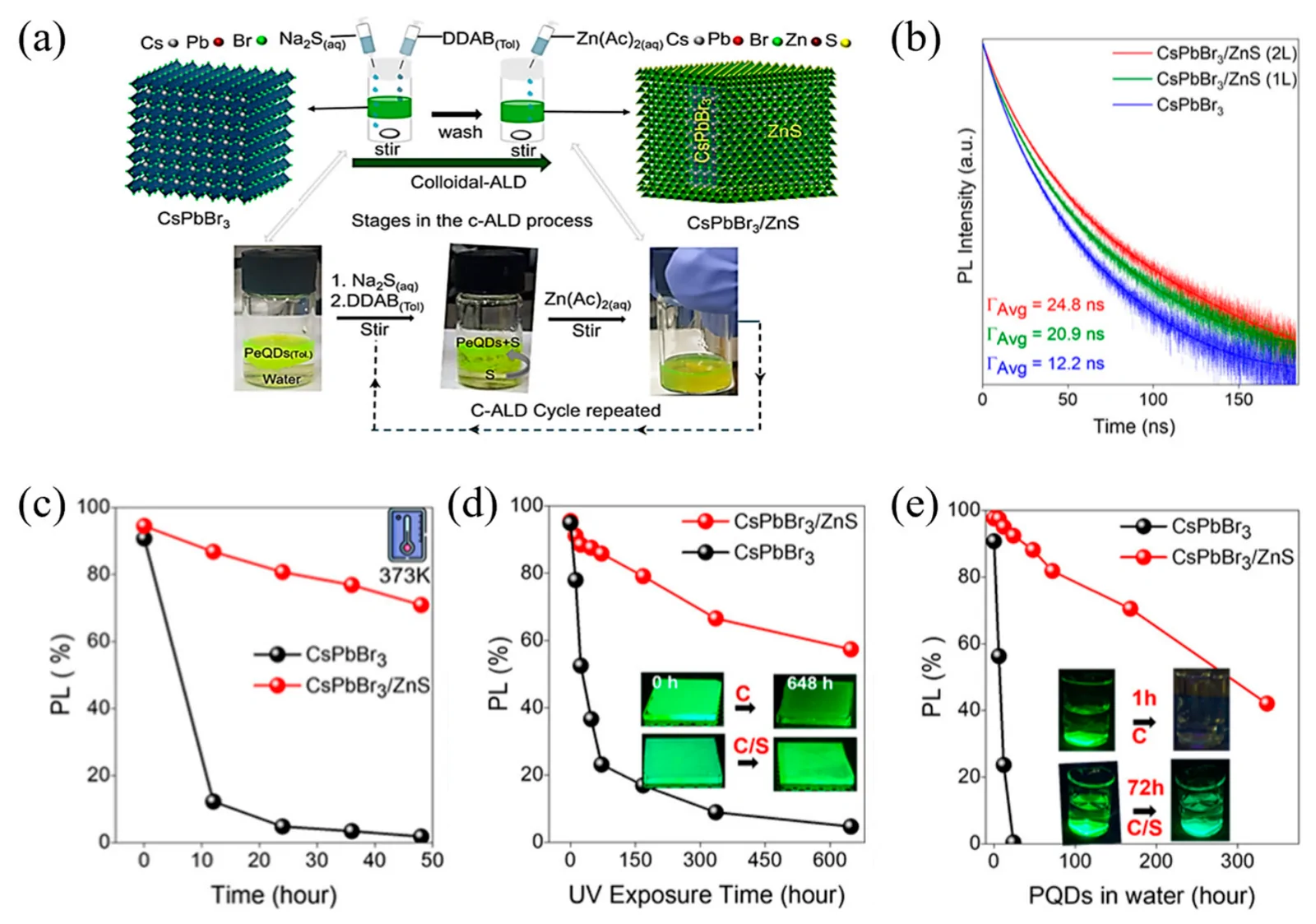
(**a**) Schematic illustration of the colloidal atomic layer deposition (c-ALD) process for the growth of ZnS shells on CsPbBr_3_ QDs. (**b**) TRPL decay profiles of pristine CsPbBr_3_ and CsPbBr_3_/ZnS core–shell QDs during c-ALD shell growth. (**c**) Thermal stability of pristine CsPbBr_3_ and CsPbBr_3_/ZnS core–shell QDs during heat treatment at 373 K, together with the corresponding temperature-dependent PL behavior. (**d**) Photostability of pristine CsPbBr_3_ and CsPbBr_3_/ZnS core–shell QDs under continuous UV exposure, presented as the evolution of relative PL intensity. (**e**) Water stability of pristine CsPbBr_3_ and CsPbBr_3_/ZnS core–shell QDs during prolonged water exposure, together with the corresponding changes in relative PL intensity and representative photographs before and after the stability test. Reprinted from Ref. [37]; copyright JACS Au 2025.

Semiconductor sulfide shells have also emerged as effective encapsulation materials. Recently, Teku et al. synthesized CsPbBr_3_/ZnS core–shell QDs using a colloidal atomic layer deposition (c-ALD) process, which enables layer-by-layer growth of a thick and conformal ZnS shell with precise thickness control, yielding shell thicknesses of approximately 0.9, 3.2, and 4.3 nm (Figure 7a). As shown in Figure 7b, TRPL measurements revealed that the average PL lifetime increased from 12.2 ns for pristine CsPbBr_3_ to 20.9 ns after one ZnS shell cycle and further to 24.8 ns after two cycles. This prolonged carrier lifetime indicates that the ZnS shell effectively passivated surface defects and suppressed non-radiative recombination. Under thermal aging at 373 K, ZnS-coated CsPbBr_3_ QDs retained approximately 75% of their initial PL intensity after 50 h, whereas pristine CsPbBr_3_ lost nearly 98% of its initial PL intensity within 10 h (Figure 7c). The ZnS shell also significantly improved photostability, with the core–shell QDs retaining approximately 65% of their initial PL intensity after 648 h of continuous UV irradiation, while pristine CsPbBr_3_ QD retained only ~10% (Figure 7d). Moreover, during water immersion, the ZnS-coated CsPbBr_3_ QDs exhibited only ~5% PL loss over the first 12 h. They maintained detectable PL even after 504 h, whereas pristine CsPbBr_3_ QD lost ~99% of its initial PL intensity within 12 h (Figure 7e). A similar sulfide-shell strategy was reported for CsPbBr_3_/CdS NCs prepared using an ion-exchange method. The CdS shell substantially improved long-term storage stability, thermal stability, and water resistance [36]. In addition, the PLQY increased from 70% for pristine CsPbBr_3_ NC to 84% after the CdS shell formation. These improvements demonstrate that CdS shell encapsulation effectively enhances the optical performance and environmental stability of PeNCs.

Overall, inorganic shell encapsulation is an effective strategy for enhancing the structural, optical, and environmental stability of pristine PeNCs. Oxide shells such as silica (SiO_2_), alumina (Al_2_O_3_), and titania (TiO_2_) primarily provide robust protection against moisture, oxygen, and thermal degradation while minimizing surface defects. Moreover, semiconductor sulfide shells such as ZnS and CdS not only provide electronic passivation through heterointerface formation but also, further suppress non-radiative recombination, thereby enhancing PL efficiency.

However, the application of inorganic shell-encapsulated PeNCs in PeLEDs requires precise control of shell thickness, core–shell interface quality, and band alignment. Trinh et al. reported that SiO_2_-encapsulated PeNCs had been restricted to nonelectrical applications, such as down-conversion LEDs, because of the insulating nature and excessive thickness of the SiO_2_ shell [38]. For CsPbBr_3_@SiO_2_ PeNCs, increasing the SiO_2_ shell thickness improved stability against polar solvents but substantially impeded charge injection, resulting in orders-of-magnitude reductions in hole mobility (from ~1.41 × 10^−3^ to ~2.08 × 10^−5^ cm^2^ V^−1^ s^−1^) and maximum luminance (from ~8600 to ~250 cd m^−2^). In contrast, an ultrathin SiO_2_ shell preserved efficient charge injection, enabling all-solution-processed PeLEDs with a current efficiency of ~5.3 cd A^−1^ [38]. They also demonstrated that A-site doping effectively suppressed lattice-strain-induced interface defects in core–shell PeNCs. In addition, Hong et al. showed that a ZnS shell with type-I band alignment and optimal energy-level matching with the hole transport layer reduced the turn-on voltage and enhanced carrier transport in PeLEDs [39]. Consequently, optimization of shell thickness, interface defect density, and band structure is necessary to achieve both environmental stability and high EL performance.

### 3.2. Polymer Shell

Polymer encapsulation offers additional advantages such as solution processability and ease of film fabrication. These polymer shells act as a protective barrier against external degradation and enhance interactions with surface ligands. They stabilize the PeNC surface by suppressing defect formation and maintaining homogeneous dispersion in solution, thus preserving the optical properties of the PeNCs.

Polystyrene (PS) is a representative polymer matrix material for PeNC encapsulation. Chen et al. developed CsPbBr_3_ NCs modified with polyacrylic acid (PAA) which were encapsulated within an in situ formed PS (iPS) matrix. The strong physical entanglement between the PAA ligands and PS chains formed a robust protective network around the CsPbBr_3_ NCs while maintaining excellent colloidal stability [40]. PAA-CsPbBr_3_ NCs@iPS retained 89% of their initial PL emission intensity under thermal conditions and showed negligible loss over 90 UV on/off cycles. Under extended UV exposure, they maintained 80% intensity after 60 h, significantly outperforming the control OA/OAm-PeNCs, which dropped to 10% after 25 h. Repeated heating–cooling cycles showed a similar contrast, bare PAA-CsPbBr_3_ NCs exhibited continuous PL degradation, whereas PAA-CsPbBr_3_ NCs@iPS maintained stable PL emission after multiple cycles. The confinement afforded by the polymer matrix effectively suppresses ligand desorption, particle aggregation, and thermally induced structural degradation. These results indicate that the hydrophobic PS shell improves moisture, thermal, and photostability.

**Figure 8:**
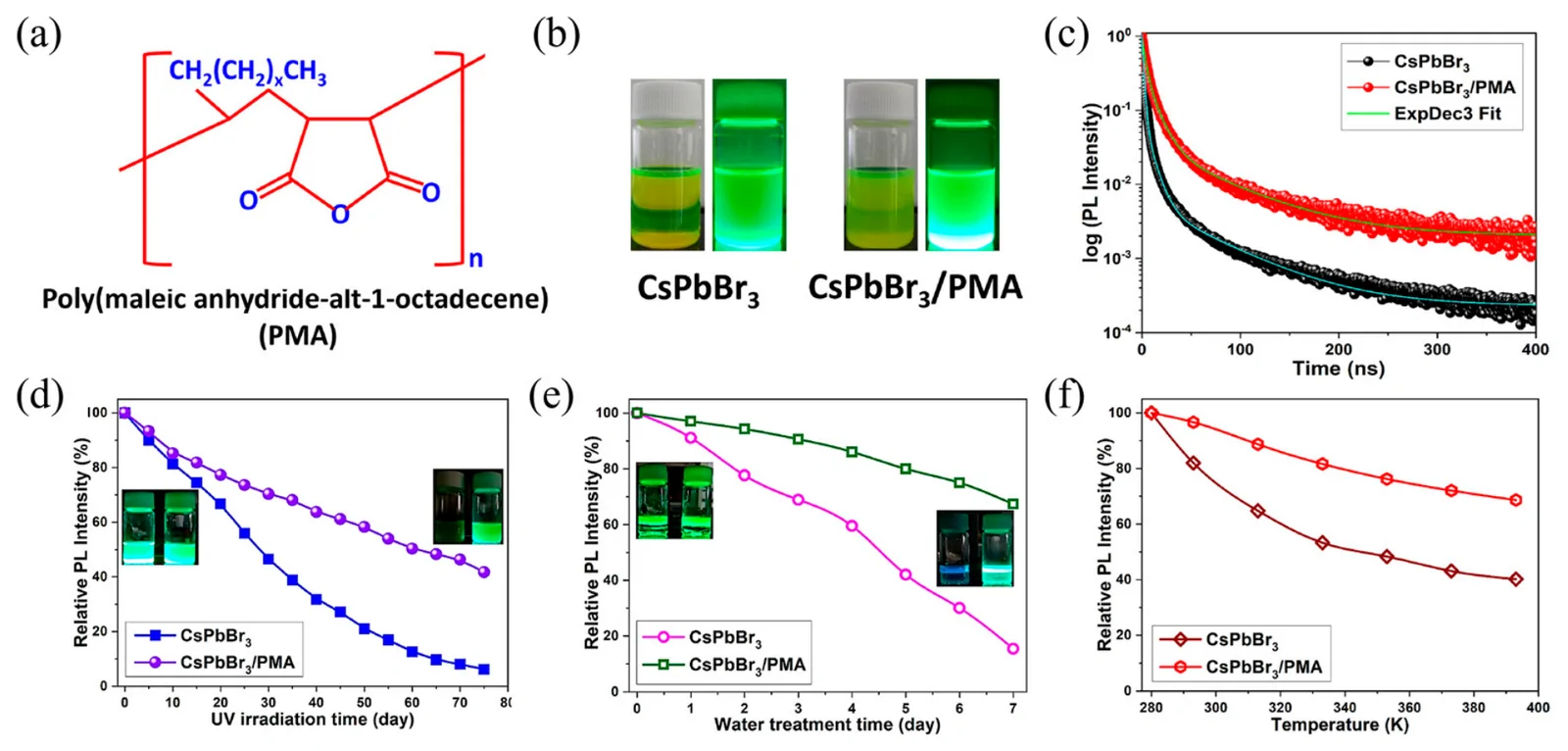
(**a**) Structure of poly (maleic anhydride-alt-1-octadecene) (PMA). (**b**) Colloidal solutions of PeNCs and PeNCs/PMA. (**c**) TRPL decay curves of PeNCs solutions. Relative PL intensity of PeNCs solutions under (**d**) UV light, (**e**) water, (**f**) different temperatures. Reprinted from Ref. [42]; copyright Heliyon 2024.

Bommireddy et al. reported CsPbBr_3_ core/poly(maleic anhydride-alt-1-octadecene) (PMA) shell NCs prepared using a modified hot-injection method (Figure 8a,b). The PMA shell coordinates with under-coordinated Pb^2+^ and Br^−^ surface sites, reducing surface defects and protecting PeNCs from environmental degradation. The PMA-encapsulated CsPbBr_3_ core exhibited a substantially higher PLQY (86.8%) than the pristine CsPbBr_3_ NCs (54.2%), together with prolonged PL lifetimes (23.5 ns) compared to the pristine (12.8 ns), indicating suppressed non-radiative recombination (Figure 8c). Moreover, after 75 days of consistent UV irradiation, CsPbBr_3_/PMA NCs retained 42.4% of their initial PL intensity compared to only 6.2% for pristine CsPbBr_3_ NCs (Figure 8d). For moisture resistance, the PMA shell maintained 68.9% PL retention after 7 days of aqueous exposure, whereas pristine CsPbBr_3_ NCs degraded to 14.1%, indicating effective prevention of water-induced aggregation (Figure 8e). Thermal stability tests over 280–293 K showed that CsPbBr_3_/PMA NCs retained 71.3% PL intensity versus 39.5% for pristine CsPbBr_3_ NCs, reflecting suppressed electron-phonon interactions and nonradiative recombination pathways (Figure 8f). They were then used as green down-conversion emitters in white LEDs. Similarly, Dong et al. developed CsPbBr_3_/linear low-density polyethylene (LLDPE) core–shell PeNCs through an in situ hot-injection process [41]. The LLDPE shell provides steric confinement and internal pressure effects that strengthen the interaction between surface ligands and the CsPbBr_3_ core while facilitating radiative recombination. The resulting CsPbBr_3_/LLDPE NCs achieved near-unity PLQY and retained 92.5% of their initial PLQY after 166 days under ambient conditions and 61.7% of their initial PL intensity after prolonged UV irradiation and were employed as color conversion layers (CCLs) for mini-LED backlight PeLEDs with a wide color gamut.

Recent studies have explored hybrid encapsulation approaches that integrate both inorganic shells and polymer matrices. By combining the robust environmental barrier provided by inorganic materials with the additional protection afforded by polymer matrices, the hybrid encapsulation structure can further effectively improve the optical performance and the long-term stability of PeNCs. One representative example is a dual-protection structure consisting of an Al_2_O_3_ shell and an outer ethylene-propylene-diene monomer (EPDM) layer [43]. The Al_2_O_3_ layer serves as an effective barrier against moisture, heat, and polar solvents, while the EPDM layer further suppresses environmental degradation and helps preserve the optical properties of the PeNCs. The CsPbBr_3_@Al_2_O_3_/EPDM NCs exhibited better water stability than CsPbBr_3_/EPDM. After 15 days of water immersion, the CsPbBr_3_@Al_2_O_3_/EPDM NCs retained 84% of its initial PL intensity, whereas CsPbBr_3_/EPDM exhibited a 42% decrease. These results confirm the synergistic protective effect of inorganic and polymer hybrid encapsulation in improving the long-term stability of PeNCs.

Overall, polymer encapsulation is an effective strategy for improving the environmental and thermal stabilities of PeNCs. In addition to serving as a hydrophobic barrier against external degradation, polymer matrices stabilize surface ligands and suppress defect formation, thereby preserving the optical performance of PeNCs under operational conditions. These stability improvements make encapsulated PeNCs suitable for practical device integration, particularly down-conversion layers in white LEDs and wide-color-gamut mini-LED backlights.

However, the electrically insulating nature of polymer matrices likely accounts for the predominant application of polymer-encapsulated PeNCs in CCLs, where charge injection is not directly required, rather than as active PeNC layers in PeLEDs. In hybrid encapsulation systems, the complex interfaces among the PeNC core, inorganic shell, and polymer matrix also require careful consideration, as interfacial defects can serve as non-radiative recombination centers and reduce PL efficiency. Consequently, the application of hybrid encapsulation in PeLEDs or CCLs requires precise control over these multiple interfaces, together with the careful selection of electrically compatible shell materials.

## 4. Crosslinking Engineering

Similar to encapsulation engineering, crosslinking blocks ambient degradation from the external environment through chemical linking of adjacent PeNCs or their surface ligands. In addition, crosslinking simultaneously suppresses internal ligand desorption and ion migration within the film. While conventional encapsulation primarily focuses on shielding perovskite cores from external exposure, the dynamic lattice behavior within the assembled film remains unmitigated. This in situ network formation addresses internal instability by generating an insoluble framework that stabilizes the entire film morphology internally.

The crosslinking strategy was initially introduced to increase the chemical resistance against solvents during subsequent multilayer depositions through solution process [44]. Because PeNC films are typically held together by dynamic surface ligands, they are susceptible to redissolution when the upper layers are deposited. Chemically linked adjacent surface ligands form an insoluble network that maintains the integrity of the film during PeLED fabrication by passivating surface defects, suppressing ion migration, and enhancing environmental stability [45,46].

**Figure 9:**
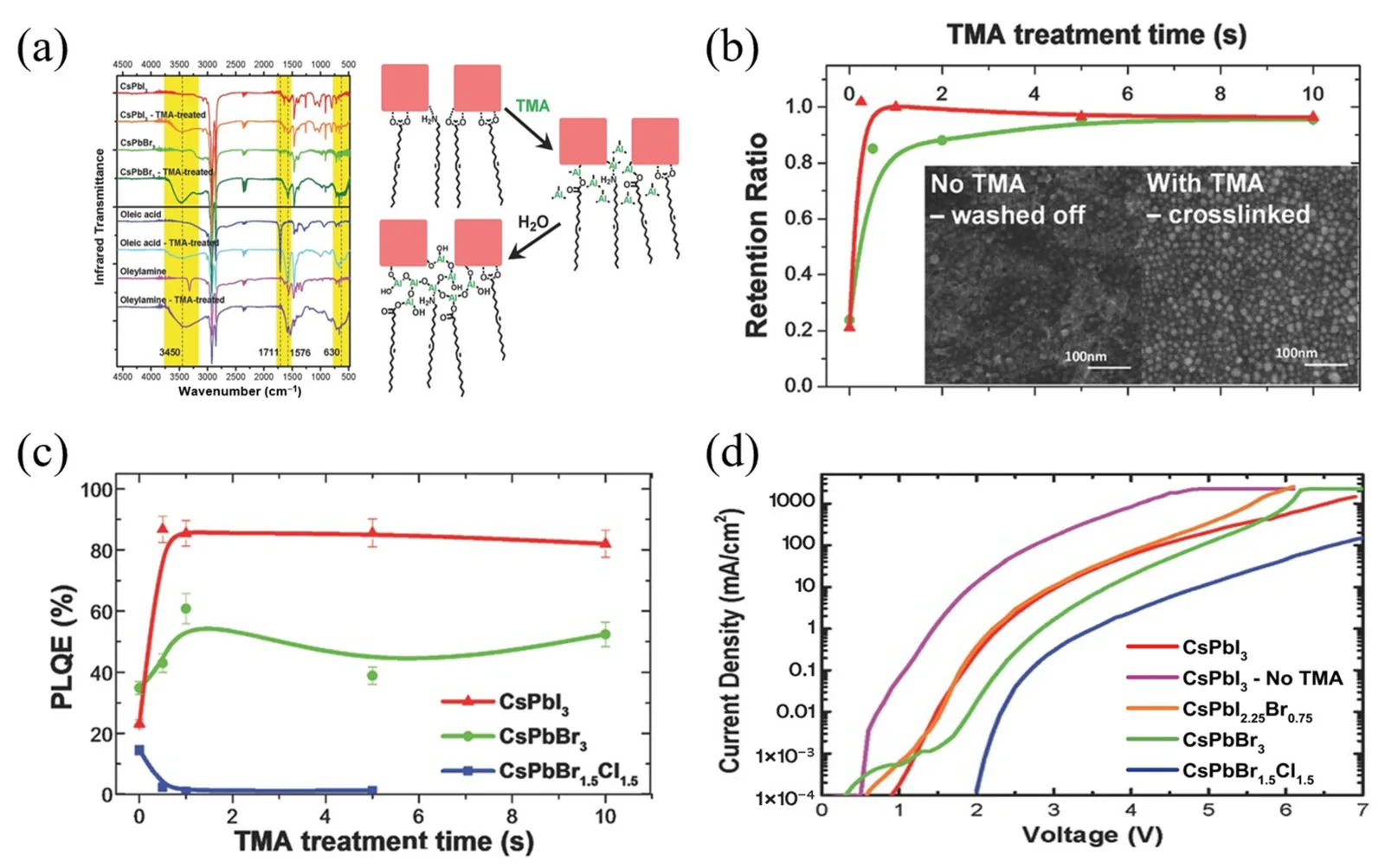
(**a**) Fourier transform infrared (FT-IR) spectra of PeNCs before and after trimethylaluminum (TMA) treatment, in which the yellow-highlighted bands indicate the O–H stretch (~3450 cm^−1^), the C=O stretch of the free carboxylic acid group of oleic acid (1711 cm^−1^), the weakened C=O stretch of the Al-coordinated carboxylate group (1576 cm^−1^), and the vibrational modes of alumina (~630 cm^−1^), and a reaction schematic of the TMA crosslinking process. (**b**) Retention ratio of TMA-treated and non-treated PeNCs upon washing with toluene (red triangles: CsPbI_3_; green circles: CsPbBr_3_) and SEM images of washed-off and crosslinked PeNCs. (**c**) PLQY (denoted as PLQE in the figure) as a function of TMA crosslinking duration. (**d**) Current density–voltage characteristics of PeLEDs based on TMA-treated CsPbX_3_ NCs with different halide compositions, compared with those based on non-treated CsPbI_3_ NCs (No TMA). Reprinted from Ref. [44]; copyright Advanced Materials 2016.

Li et al. first demonstrated the effect of crosslinking in PeNCs by introducing a universal vapor-phase crosslinking process based on trimethylaluminum (TMA) for CsPbX_3_ (X = Cl, Br, I) NC films. TMA vapor reacts with the carboxyl and amino groups of the native ligands and subsequently hydrolyzes with ambient moisture to form an AlO_x_ network linked to the PeNC surface, forming an external network without significantly disturbing the original PeNC arrangement. As shown in Figure 9a, the infrared spectroscopic changes in the film as a function of the TMA exposure time were consistent with this reaction pathway. With vapor exposure times shorter than 0.5 s, nearly 100% film retention was observed during subsequent toluene washing, and short TMA treatment increased the PLQYs of both CsPbI_3_ and CsPbBr_3_ PeNCs under optimized conditions (Figure 9b,c). PeLEDs based on TMA-crosslinked CsPbI_3_ exhibited more than a tenfold increase in current density at the same or higher luminance than pristine PeLEDs, indicating that crosslinking effectively reduces the leakage current and improves the charge-injection efficiency (Figure 9d).

Crosslinking has also been applied at the molecular level, focusing on the direct modification of surface ligands or the introduction of functional small molecules. Sun et al. incorporated crosslinkable vinyl groups into the termini of surface ligands without relying on external binder materials [47]. UV-induced covalent bonding between ligands produces a surface-linked network that maintains over 80% of the initial PLQY after three solvent-washing cycles and preserves colloidal dispersibility for over four months in solution. Similarly, Han et al. introduced methylene-bis-acrylamide (MBA), a small molecule with dual acrylamide groups, to form a covalently crosslinked matrix at the film interface and directly address ion migration, an intrinsic degradation pathway under high electric fields. MBA crosslinking increased the ligand-desorption energy from 0.54 to 0.92 eV and the diffusion activation energy (E_a_) for halide-ion migration from 0.21 to 0.50 eV, thereby suppressing ion movement. This reduced the formation of nonradiative recombination centers during operation, enabling a PeLED with a T_50_ of 208 h at the initial luminance of 100 cd/m^2^ [48].

**Figure 10:**
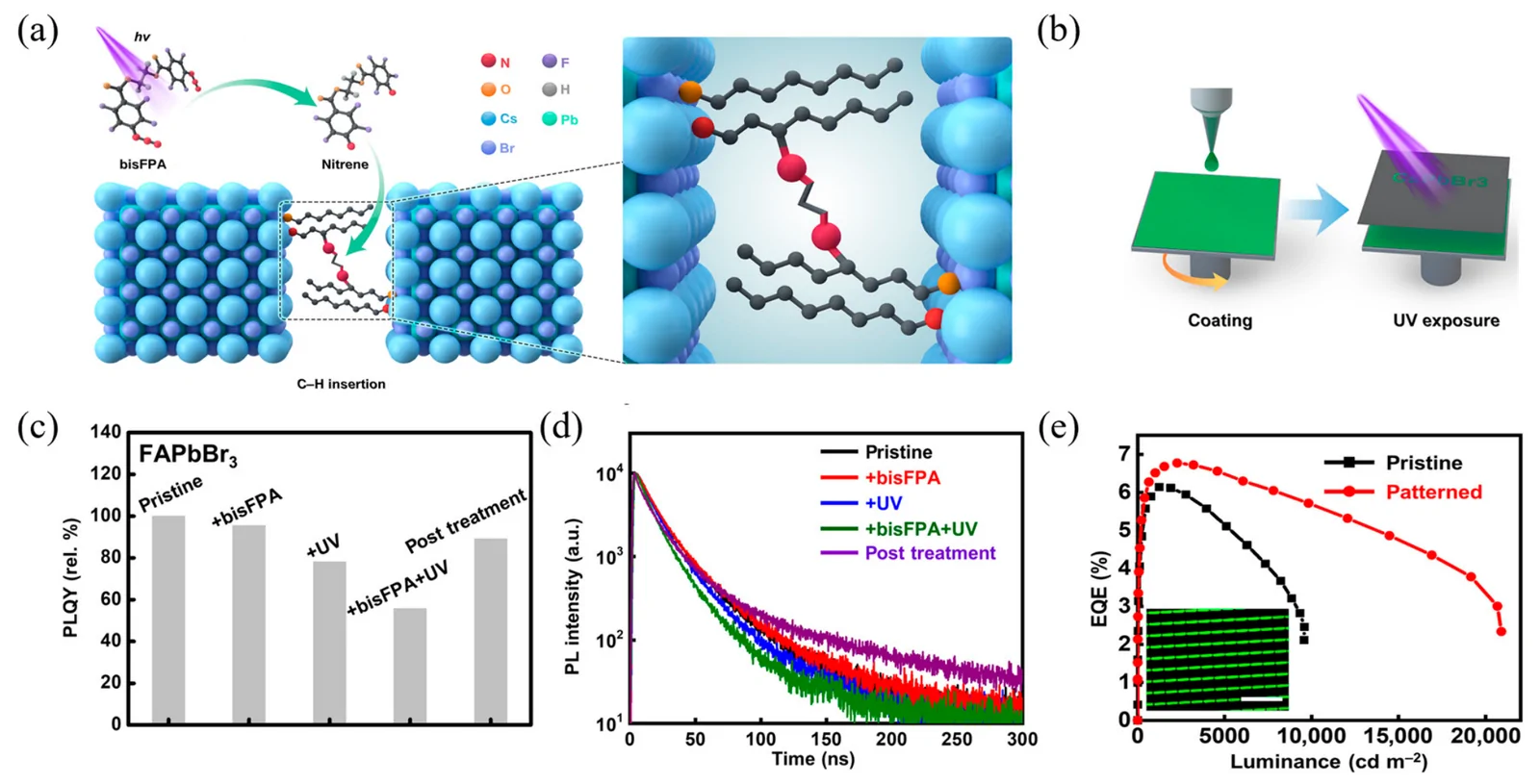
Schematic of (**a**) photo-crosslinking chemistry and (**b**) crosslinked film fabrication procedure of PeNC spin coating and UV exposure. (**c**) Relative PLQYs and (**d**) PL decay curves of FAPbBr_3_ NC films. (**e**) EQE-luminance graph and image of a pixelated PeLED. Reprinted from Ref. [49]; copyright Science Advances 2022.

Other studies have used UV-driven radical chain reactions for crosslinking to increase solvent resistance while preserving the native ligand environment. Liu et al. reported a strategy in which the photoactive additive of bis(fluorophenyl azide) (bisFPA) generates nitrene radicals under UV exposure (Figure 10a,b). These radicals undergo nonspecific C–H insertion into the alkyl chains of the native ligands, forming a covalent organic network without requiring an additional ligand-exchange step. The absolute PLQYs of the pristine, bisFPA-added (10 wt%), UV-exposed, and post-treatment-applied FAPbBr_3_ NC films were 86%, 82% (96%), 67% (78%), and 48% (56%), respectively (Figure 10c). Adding 10 wt% bisFPA resulted in a negligible decrease in the PLQY due to electronic energy level alignment, where the highest occupied molecular orbital (HOMO) and lowest unoccupied molecular orbital (LUMO) of bisFPA lie outside the conduction band minimum (CBM) and valence band maximum (VBM) of the PeNCs, suggesting that bisFPA does not act as a carrier or exciton trap site. The bisFPA-added films and UV exposure films showed slightly reduced average lifetimes (~6.6 and 7.1 ns, respectively, versus 7.5 ns for pristine films), while those post-treatment-applied showed a notably shorter average PL lifetime of 4.6 ns (Figure 10d). Nevertheless, the post-patterning surface treatment effectively replaced the crosslinker moieties. This introduces additional Br anions, supporting a remarkable recovery of the PLQY and a longer average PL lifetime characteristic of better surface passivation. This approach enabled stabilization without additional photoresist processing, yielding sub-5 μm patterning, an EQE of 6.8%, and pixelated PeLEDs with a luminance value above 20,000 cd/m^2^ (Figure 10e).

Similarly, Shin et al. partially replaced PeNC surface ligands with terminal olefin ligands, 9-decenoic acid (DeA), and used light-induced ligand polymerization under UV irradiation within the presence of a photoinitiator [50]. The resulting carbon radical chain reaction linked adjacent ligand shells, improving resistance to nonpolar solvents such as hexane and octane, suppressing redissolution of the emissive layer, and remaining compatible with the subsequent coating of upper charge-transport layers such as ZnO. Furthermore, Xu et al. introduced a multidentate crosslinking strategy using α-lipoic acid (LA), a small molecule containing a carboxyl group and a disulfide loop [51]. LA molecules first passivate the surface defects and then undergo spontaneous, reversible ring-opening polymerization under mild thermal treatment to form a poly(LA) network at the interface. This process mitigated lattice deformation under Joule heating, reduced the root-mean-square roughness (RMS) to 3.8 nm, and suppressed Br– ion migration toward the hole transport layer (HTL), as confirmed by time-of-flight secondary ion mass spectrometry (TOF-SIMS) depth profile. The resulting PeLEDs achieved an EQE of 25.6%, a peak luminance of 56,798 cd/m^2^, a 6.7-fold improvement in T_50_ (6.1 h) under device operation, and over 1340 h of stable operation at 100 cd/m^2^.

However, such linear ligand-crosslinking methods cannot completely passivate undercoordinated surface defects or fully cover the voids between PeNCs. Therefore, multisite crosslinking strategies have been introduced to further improve defect passivation effect and three-dimensional network formation [52]. Ma et al. developed a three-dimensional (3D) crosslinked network via UV-induced thiol–ene click reactions using the multisite crosslinkers pentaerythritol tetra(3-mercaptopropionate) (PETMP) and pentaerythritol allyl ether (PAE). This system forms a dense and hydrophobic protective layer at the grain boundaries and film surface, effectively obstructing moisture ingress and improving environmental stability. Additionally, this 3D organic network stabilizes the lattice, thereby reducing the electron–phonon interaction strength (Γ_LO_) from 207 to 167 meV and increasing the halide-ion migration E_a_ from 106 to 209 meV. As a result, a T_50_ of 47 min at 1000 cd/m^2^ was reported, along with aqueous photolithography and the fabrication of 80 μm micropixel arrays. Similarly, Lu et al. introduced a multifunctional crosslinker, 1,4-bis(3-mercaptobutyryloxy) butane (BD1), in which thiol groups passivate under-coordinated surface Pb^2+^ sites while also participating in thiol–ene click crosslinking with OA ligands [53]. This dual functionality ensures structural cohesion without sacrificing electronic property, maintaining a high PL intensity after octane exposure, and yielding excellent air-storage stability (29.8% PLQY retention after 14 days).

These 3D crosslinking structures address lattice deformation and ion migration, although the insulating character of the organic matrix must be balanced against the electronic coupling between PeNCs and the charge transport in PeLEDs. In parallel, surface-localized direct energy irradiation using a native ligand shell has been investigated as an additive-free strategy [54]. Leo et al. used electron-beam irradiation to selectively crosslink the native OA/OAm ligand shell at the surface of PeNC films. Controlling the electron-beam penetration limited the crosslinking depth to the immediate surface boundary of the film, while the underlying emissive region remained unaffected. Within an optimized low-dose range (500–1000 μC/cm^2^), the crosslinked surface layer showed strong hydrophobic properties and reduced moisture ingress and lattice coalescence. Under long-term ambient exposure, owing to the formation of a stabilizing ligand network that suppresses ion diffusion, the PL intensity of these films gradually increased to 180–250% of its initial value after three weeks. In contrast, the control film degraded completely over the same period. This crosslinked surface layer also suppressed ion diffusion, enabling the stacking of I-based layers on Br-based layers without anion exchange.

In summary, these diverse crosslinking strategies, from external inorganic networks to molecular-level and surface-localized networks, effectively improve the structural and chemical robustness of PeNC films. The implementation of these multi-dimensional crosslinking approaches successfully suppresses ligand desorption and retards ion migration under external factors. These advantages make crosslinking a practical approach, presenting a pathway toward the high stability of PeNC films or long-term operational reliability in PeLEDs.

While crosslinking offers these significant advantages, the trade-off between enhanced PeNC film stability and charge transport in PeLEDs must also be considered. Crosslinking can retard PeLED degradation by suppressing ion migration and defect formation; however, nonconductive network components or excessive crosslinking may impede interparticle charge transport and carrier injection into the PeNC layer. For instance, in inkjet-printed PeLEDs employing BD1 as a crosslinker, the EQEs increased from 13.72% to 18.63% as the BD1 concentration was increased to 2.0 wt% but decreased to 10.91% at 8.0 wt% [23]. The reduction in EQE at higher BD1 concentrations was attributed to the decreased conductivity arising from the insulating properties of BD1. Therefore, the density of crosslinkers and the electrical properties of the resulting network should be carefully optimized to suppress ion migration and defect-related non-radiative recombination while preserving efficient carrier injection and transport.

Table 1 summarizes the PeNC stabilization strategies reviewed in Section 2, Section 3 and Section 4, along with their key materials, advantages, and reported PeLED performance (EQEs and T_50_). As discussed in Section 2.1, Section 3.1, Section 3.2 and Section 4, each strategy has intrinsic limitations. Ligand engineering requires balancing the use of excess insulating ligands against defect formation resulting from excessive ligand stripping [24]. In contrast, encapsulation and crosslinking frequently introduce thick inorganic shells and nonconductive organic networks, respectively [23,38,48]. In each case, these insulating barriers impede carrier injection and transport in PeLEDs. Therefore, the enhancement of PeNC stability and the electrical properties of the resulting films should be evaluated simultaneously when selecting an optimal stabilization strategy.

## 5. Conclusions and Outlook

Ligand engineering, encapsulation, and crosslinking strategies address the instability of PeNCs at the ligand–surface interface, particle scale, and film, respectively. Ligands with high coordination affinity to surface Pb^2+^ sites passivate halide vacancies and reduce non-radiative recombination, improving the efficiency and operational lifetime of PeLEDs. Encapsulation with inorganic shells or polymer matrices protects PeNCs against moisture, oxygen, and heat, making them suitable for color-conversion applications, whereas crosslinking improves the solvent resistance of PeNC films and suppresses ion migration in PeLEDs. However, as described at the end of Section 4, each strategy has intrinsic limitations. Excess insulating ligands, thick encapsulation layers, and highly crosslinked organic networks can hinder charge injection and carrier transport in PeLEDs. Therefore, the ligand or crosslinker concentration, the number of washing steps, and the shell thickness should be carefully optimized. Each strategy and its combinations should also be evaluated in PeLEDs because operational stability cannot be inferred from PL performance alone.

In addition, the operational lifetime of PeLEDs is affected by device-level factors, such as the charge imbalance arising from interfacial defects or energy barriers and the degradation induced by heat or ion migration. Carrier injection and recombination in operating PeLEDs depend not only on the intrinsic properties of PeNCs but also on interfacial charge injection and charge balance within the PeNC layer. Therefore, device engineering, including interfacial engineering and charge carrier dynamics, should be studied together with material engineering. Further studies are also needed to establish a direct correlation between the operational lifetime of PeLEDs and the stability of the PeNC solutions and films.

Several bottlenecks also remain for the practical application of PeLEDs in displays. In blue PeLEDs, the small PeNCs required for strong quantum confinement have a high surface-to-volume ratio, which raises the surface defect density and accelerates degradation [39]. The surface passivation strategies, together with the suppression of interfacial defects during film formation, are expected to improve the stability of blue PeLEDs. For industrial manufacturing, improved intrinsic stability of PeNCs can help maintain the ink quality during storage and coating [55,56]. In addition, some of the crosslinking strategies reviewed in Section 4 were compatible with photolithography and inkjet printing, which are promising for pixel patterning in displays. Scalable synthesis and deposition processes should be further developed alongside material stabilization for the commercialization of PeLEDs [55,56].

Replacing Pb with less toxic elements such as Sn and Bi is another important issue. Although the high-performance PeLEDs are based on Pb-containing PeNCs, ligand engineering and surface passivation have also been reported to improve the stability of Sn- and Bi-based PeNCs [57,58,59,60], indicating that the strategies discussed in this review can be extended to Pb-free compositions.

Finally, standardized lifetime testing protocols are required for evaluating PeLEDs. Because specific protocols for PeLEDs have not yet been established and PeLEDs show an initial luminance overshoot and ion-migration-induced changes in luminance during operation, the measurement conditions, the measurement duration, and the luminance–time curves should be reported together with T_50_ [8,14]. By comparing the strengths and limitations of each strategy, this review outlines the design criteria for stable PeNCs and identifies the remaining gaps to guide the future development of commercially available PeLEDs.

## Figures and Tables

**Table 1:** Summary of PeNC stabilization strategies and their applications.

Strategy	Materials	Advantages	Device Performance (EQEs, T_50_)	Ref.
Ligand engineering	OPA-treated CsPbBr_3_ PeNCs	Suppresses ligand desorption and maintains PLQY during repeated washing	-	[20]
	DOAB-treated CsPbBr_3_ PeNCs	Compensates halide vacancies; increases PLQY and improves ambient stability	-	[21]
	DDeAB-treated CsPbBr_3_ PeNCs	Compensates halide vacancies; the shorter ligand shell improves electronic coupling between PeNCs	EQE = 9.71%	[21]
	ASC18-treated CsPbBr_3_ PeNC films	Multidentate surface protection through the chelate effect; increases PLQY	-	[22]
	Lecithin-treated CsPbBr_3_ PeNC films	Smooth, defect-free film with uniform morphology; lowest ASE threshold	-	[22]
	HFPA-treated CsPb(Br/Cl)_3_ PeNCs	Suppresses defects and ion migration through multi-site binding; the short ligand preserves carrier injection and transport	EQE = 14.8%, T_50_ = 341.6 s (control: 27.2 s)	[23]
	Zinc-octanoate-treated CsPbBr_3_ PeNCs	Regulates surface acid–base equilibrium and suppresses nanoplatelet formation; increases PLQY and average lifetime	-	[24]
	PbBr_2_-treated amine-free CsPbBr_3_ PeNCs	Passivates Br^−^ vacancies and under-coordinated Pb^2+^; maintains PLQY during long-term storage	-	[25]
	TEAC-treated CsPbI_3_ PeNCs	Dual-site anchoring suppresses trap states; maintains high PLQY after intensive purification	EQE = 17.3%, T_50_ = ~10 h (control: 5 h)	[28]
	2-ThSA-treated CsPbI_3_ PeNCs	Higher binding energy than OA/OAm; increases PLQY and maintains it during storage	-	[26]
	4-FBSA-treated CsPb(Br/I)_3_ PeNCs	Passivates surface defects and improves hole injection; increases PLQY	EQE = 21.8% (control: 11.3%)	[29]
Encapsulation	CsPbBr_3_@SiO_2_ PeNCs (one-pot synthesis)	Isolates PeNCs from moisture and oxygen; increases average PL lifetime	-	[32]
	PE-ALD SiO_2_-coated CsPbBr_3_ PeNC films	Conformal SiO_2_ layer; maintains PL under UV irradiation and water immersion	-	[31]
	CsPbBr_3_/ZrO_2_ Janus heterostructure PeNCs	Type-I heterostructure confines carriers; increases PLQY and improves polar-solvent resistance	-	[34]
	CsPbBr_3_/ZnS core–shell PeNCs	Thick conformal shell; maintains PL under prolonged UV irradiation and heating	-	[33]
	CsPbBr_3_/CdS core–shell PeNCs	Increases PLQY; improves long-term storage, thermal, and water stability	-	[35]
	PAA-modified CsPbBr_3_ PeNCs@iPS	Suppresses ligand desorption and particle aggregation; maintains PL under UV exposure	-	[37]
	CsPbBr_3_/PMA core–shell PeNCs	Increases PLQY; maintains PL under UV exposure and in water	-	[36]
	CsPbBr_3_/LLDPE core–shell PeNCs	Near-unity PLQY, maintained during long-term ambient storage	-	[38]
	CsPbBr_3_@Al_2_O_3_ /EPDM hybrid PeNCs	Maintains PL during water immersion	-	[39]
Crosslinking	TMA-crosslinked CsPbX_3_ (X = Cl, Br, I) PeNC films	Retains the film after toluene washing with brief TMA exposure; increases PLQY under optimized conditions	-	[40]
	UV-crosslinked MAPbBr_3_ PeNCs	Maintains PLQY after repeated washing and colloidal dispersibility during long-term storage	-	[43]
	MBA-crosslinked PeNCs	Raises ligand-desorption energy and halide-ion migration activation energy	T_50_ = 208 h (@ 100 cd/m^2^)	[44]
	bisFPA-crosslinked FAPbBr_3_ PeNCs	Solvent-resistant network without photoresist processing; enables fine patterning	EQE = 6.8%	[45]
	DeA-crosslinked PeNCs	Improves resistance to nonpolar solvents (hexane, octane) and allows subsequent ZnO coating	-	[46]
	LA-crosslinked CsPbBr_3_ PeNCs	Mitigates lattice deformation under Joule heating and suppresses Br^−^ migration toward the HTL; forms a smooth film	EQE = 25.6%, T_50_ = 6.1 h	[47]
	PETMP/PAE- crosslinked PeNCs (3D network)	Maintains PL intensity after octane exposure and retains PLQY in air	T_50_ = 47 min (@ 1000 cd/m^2^)	[48]
	BD1- crosslinked PeNCs	Combines surface Pb^2+^ passivation with crosslinking	-	[49]
	Electron-beam- crosslinked PeNC films	Increases PL intensity during ambient storage; suppresses ion diffusion, enabling I-based layers on Br-based layers	-	[50]

## Data Availability

No new data were created or analyzed in this study. Data sharing is not applicable to this article.

## Abbreviations

The following abbreviations are used in this manuscript:

PeNCs	Perovskite nanocrystals
LEDs	Light-emitting diodes
PL	Photoluminescence
PeLEDs	Perovskite light-emitting diodes
FWHM	Full width at half maximum
PLQY	Photoluminescence quantum yield
OA	Oleic acid
OAm	Oleylamine
OPA	Octylphosphonic acid
TGA	Thermogravimetric analysis
QAB	Quaternary ammonium bromide
DOAB	Dioctyldimethylammonium bromide
DDeAB	Didecyldimethyl ammonium bromide
EQE	External quantum efficiency
SEM	Scanning electron microscopy
ASC18	3-(N,N-dimethyloctadecylammonio)-propanesulfonate
DDAB	Didodecyldimethylammonium bromide
ASE	Amplified spontaneous emission
HFPA	(1H,1H,2H,2H-heptadecafluorodec-1-yl)phosphonic acid
EL	Electroluminescence
QDs	Quantum dots
TEAC	2-thiophenethylamine chloride
TRPL	Time-resolved photoluminescence
DFT	Density functional theory
2-ThSA	Thiophene-2-sulfonamide
E_b_	Binding energy
fs-TA	Femtosecond transient absorption
4-FBSA	4-fluorobenzenesulfonamide
HODs	Hole-only devices
PE-ALD	plasma-enhanced atomic layer deposition
UV	Ultraviolet
c-ALD	Colloidal atomic layer deposition
PS	Polystyrene
PPA	Polyacrylic acid
iPS	In situ formed PS
PMA	Poly(maleic anhydride-alt-1-octadecene)
LLDPE	Linear low-density polyethylene
CCLs	Color conversion layers
EPDM	Ethylene-propylene-diene monomer
FT-IR	Fourier transform infrared
TMA	Trimethylaluminum
MBA	Methylene-bis-acrylamide
E_a_	Activation energy
T_50_	Half-lifetime
bisFPA	Bis(fluorophenyl azide)
HOMO	Highest occupied molecular orbital
LUMO	Lowest unoccupied molecular orbital
CBM	Conduction band minimum
VBM	Valence band maximum
DeA	9-decenoic acid
LA	α-Lipoic acid
RMS	Root-mean-square roughness
HTL	Hole transport layer
TOF-SIMS	Time-of-flight secondary ion mass spectrometry
PETMP	Pentaerythritol tetra(3-mercaptopropionate)
PAE	Pentaerythritol allyl ether
3D	Three-dimensional
Γ_LO_	Electron-phonon interaction strength
BD1	1,4-bis(3-mercaptobutyryloxy)butane

## References

[1] Liu Y., Ma Z., Zhang J., He Y., Dai J., Li X., Shi Z., Manna L. (2025). Light-Emitting Diodes Based on Metal Halide Perovskite and Perovskite Related Nanocrystals. Adv. Mater..

[2] Zhao Y., Choi B.H., Kim E., Zeng Q., Lee T.W. (2025). Advancing Perovskite Nanocrystals for High-Resolution LED Displays. Commun. Mater..

[3] Aftabuzzaman M., Hong Y., Jeong S., Levan R., Lee S.J., Choi D.H., Lee K. (2025). Colloidal Perovskite Nanocrystals for Blue-Light-Emitting Diodes and Displays. Adv. Sci..

[4] Liu A., Bi C., Li J., Zhang M., Cheng C., Binks D., Tian J. (2023). High Color-Purity and Efficient Pure-Blue Perovskite Light-Emitting Diodes Based on Strongly Confined Monodispersed Quantum Dots. Nano Lett..

[5] Yun H.S., Noh K., Kim J., Noh S.H., Kim G.H., Lee W., Na H.B., Yoon T.S., Jang J., Kim Y. (2020). CsPbBr_3_ Perovskite Quantum Dot Light-Emitting Diodes Using Atomic Layer Deposited Al_2_O_3_ and ZnO Interlayers. Phys. Status Solidi RRL.

[6] Ahn S., Park M.H., Jeong S.H., Kim Y.H., Park J., Kim S., Kim H., Cho H., Wolf C., Pei M. (2019). Fine Control of Perovskite Crystallization and Reducing Luminescence Quenching Using Self-Doped Polyaniline Hole Injection Layer for Efficient Perovskite Light-Emitting Diodes. Adv. Funct. Mater..

[7] Cho H., Jeong S.-H., Park M.-H., Kim Y.-H., Wolf C., Lee C.-L., Hyuck Heo J., Sadhanala A., Myoung N., Yoo S. (2015). Overcoming the Electroluminescence Efficiency Limitations of Perovskite Light-Emitting Diodes. Science.

[8] Jeong S.H., Park J., Han T.H., Zhang F., Zhu K., Kim J.S., Park M.H., Reese M.O., Yoo S., Lee T.W. (2020). Characterizing the Efficiency of Perovskite Solar Cells and Light-Emitting Diodes. Joule.

[9] Park G.J., Shin K.B., Park M.H. (2025). Efficient and Stable Pure-Blue Perovskite Light-Emitting Diodes for High-Color-Purity Advanced Displays. ACS Appl. Electron. Mater..

[10] Kim D.H., Woo S.J., Huelmo C.P., Park M.H., Schankler A.M., Dai Z., Heo J.M., Kim S., Reuveni G., Kang S. (2024). Surface-Binding Molecular Multipods Strengthen the Halide Perovskite Lattice and Boost Luminescence. Nat. Commun..

[11] Wu Y., Chen D., Zou G., Liu H., Zhu Z., Rogach A.L., Yip H.L. (2025). Strategies for Stabilizing Metal Halide Perovskite Light-Emitting Diodes: Bulk and Surface Reconstruction of Perovskite Nanocrystals. ACS Nano.

[12] Yuan B., Zhong H., Shan Q., Hu T., Wu X., Cai X., Huang M., Zeng H. (2025). Recent Advances in Stability Enhancement of Metal Halide Perovskite Light-Emitting Diodes. Chem. Commun..

[13] Yang J.N., Wang J.J., Yin Y.C., Yao H.-B. (2023). Mitigating Halide Ion Migration by Resurfacing Lead Halide Perovskite Nanocrystals for Stable Light-Emitting Diodes. Chem. Soc. Rev..

[14] Kim H., Kim J.S., Heo J.M., Pei M., Park I.H., Liu Z., Yun H.J., Park M.H., Jeong S.H., Kim Y.H. (2020). Proton-Transfer-Induced 3D/2D Hybrid Perovskites Suppress Ion Migration and Reduce Luminance Overshoot. Nat. Commun..

[15] Udayabhaskararao T., Houben L., Cohen H., Menahem M., Pinkas I., Avram L., Wolf T., Teitelboim A., Leskes M., Yaffe O. (2018). A Mechanistic Study of Phase Transformation in Perovskite Nanocrystals Driven by Ligand Passivation. Chem. Mater..

[16] Hwang H., Kim M., Kim S., Kim Y., Han M., Bootharaju M.S., Min M., Hyeon T., Kim B.H. (2025). Ligand-Controlled Fe_3_O_4_ nanoparticles as Reusable Adsorbents for Dye Removal in the Chemical Recycling of Coloured Polyester Textiles. Nanoscale.

[17] Xu M., Ku K.H., Lee Y.J., Kim T., Shin J.J., Kim E.J., Choi S.H., Yun H., Kim B.J. (2021). Effect of Polymer Ligand Conformation on the Self-Assembly of Block Copolymers and Polymer-Grafted Nanoparticles within an Evaporative Emulsion. Macromolecules.

[18] Fiuza-Maneiro N., Sun K., López-Fernández I., Gómez-Graña S., Müller-Buschbaum P., Polavarapu L. (2023). Ligand Chemistry of Inorganic Lead Halide Perovskite Nanocrystals. ACS Energy Lett..

[19] Hills-Kimball K., Yang H., Cai T., Wang J., Chen O. (2021). Recent Advances in Ligand Design and Engineering in Lead Halide Perovskite Nanocrystals. Adv. Sci..

[20] Tan Y., Zou Y., Wu L., Huang Q., Yang D., Chen M., Ban M., Wu C., Wu T., Bai S. (2018). Highly Luminescent and Stable Perovskite Nanocrystals with Octylphosphonic Acid as a Ligand for Efficient Light-Emitting Diodes. ACS Appl. Mater. Interfaces.

[21] Park J.H., Lee A.Y., Yu J.C., Nam Y.S., Choi Y., Park J., Song M.H. (2019). Surface Ligand Engineering for Efficient Perovskite Nanocrystal-Based Light-Emitting Diodes. ACS Appl. Mater. Interfaces.

[22] Milanese S., De Giorgi M.L., Morello G., Bodnarchuk M.I., Anni M. (2025). Role of the Capping Ligand in CsPbBr_3_ Nanocrystals Amplified Spontaneous Emission Properties. ACS Appl. Nano Mater..

[23] Maimaitizi H., Ågren H., Chen G. (2026). Multifunctional Ligand Engineering Enables High-Performance CsPb(Br/Cl)_3_ Nanocrystals toward Efficient and Stable Pure-Blue Perovskite LEDs. Light Sci. Appl..

[24] Dai J., Roshan H., De Franco M., Goldoni L., De Boni F., Xi J., Yuan F., Dong H., Wu Z., Di Stasio F. (2024). Partial Ligand Stripping from CsPbBr_3_ Nanocrystals Improves Their Performance in Light-Emitting Diodes. ACS Appl. Mater. Interfaces.

[25] Li L., Zheng W., Wan Q., Liu M., Zhang Q., Zhang C., Yan R., Feng X., Kong L. (2021). CsPbBr_3_ Nanocrystal Light-Emitting Diodes with Efficiency up to 13.4% Achieved by Careful Surface Engineering and Device Engineering. J. Phys. Chem. C.

[26] Zhang Z.H., Shen W.S., Wang Y.K., Liao L.S. (2026). Bridging Synthesis and Device Performance in Perovskite Quantum Dot Light-Emitting Diodes. Adv. Mater..

[27] Chen Y., Nan M., He Y., Lu S., Shen W., Cheng G., Chen S., Huang W. (2024). Z-Type Ligand Enables Efficient and Stable Deep-Blue Perovskite Light-Emitting Diodes. ACS Appl. Mater. Interfaces.

[28] Akhil S., Dutt V.G.V., Singh R., Mishra N. (2022). Post-Synthesis Treatment with Lead Bromide for Obtaining Near-Unity Photoluminescence Quantum Yield and Ultra-Stable Amine-Free CsPbBr_3_ Perovskite Nanocrystals. J. Phys. Chem. C.

[29] Li C., Yao J., Xu L., Yang Z., Wang S., Cai B., Wang J., Fan W., Wang Y., Song J. (2024). Multifunctional Ligand-Manipulated Luminescence and Electric Transport of CsPbI_3_ Perovskite Nanocrystals for Red Light-Emitting Diodes. Chem. Eng. J..

[30] Li J., Hu X., Feng Y., Chen X., Ye J., Wang X., Yang C., Zhang D., Cai Q., He H. (2025). Highly Efficient Pure Red Light-Emitting Diodes Enabled by Multifunctional Ligand-Coordinated CsPbI_3_ Quantum Dots. Nano Energy.

[31] Feng Y., Zhu M., Cao Q., Li H., Zhu X., Lyu X., Dai X., He H., Ye Z. (2024). P-π Conjugated L-Type Ligand for Pure-Red Perovskite Light-Emitting Diodes. ACS Energy Lett..

[32] Shirke Y.M., Yu Y.J., Chung J.W., Cho S.J., Kwon S.J., Hong S.U., Jeon J.D. (2024). Hydrophobic Hollow Fiber Composite Membranes Based on Hexadecyl-Modified SiO_2_ Nanoparticles for Toluene Separation. J. Environ. Chem. Eng..

[33] Zhong Q., Cao M., Hu H., Yang D., Chen M., Li P., Wu L., Zhang Q. (2018). One-Pot Synthesis of Highly Stable CsPbBr_3_@SiO_2_ Core-Shell Nanoparticles. ACS Nano.

[34] Jing Y., Merkx M.J.M., Cai J., Cao K., Kessels W.M.M., Mackus A.J.M., Chen R. (2020). Nanoscale Encapsulation of Perovskite Nanocrystal Luminescent Films via Plasma-Enhanced SiO_2_ Atomic Layer Deposition. ACS Appl. Mater. Interfaces.

[35] Liu H., Tan Y., Cao M., Hu H., Wu L., Yu X., Wang L., Sun B., Zhang Q. (2019). Fabricating CsPbX_3_-Based Type I and Type II Heterostructures by Tuning the Halide Composition of Janus CsPbX_3_/ZrO_2_ Nanocrystals. ACS Nano.

[36] Hu J., Ning S., Hao C., Ren Z., Li C., Wang F., Dong G., Yue G., Guan L., Li X. (2024). Synthesis and Performance Optimization of CsPbBr_3_/CdS Core/Shell Lead Halide Perovskite Nanocrystals by an Ion Exchange Method. Opt. Express.

[37] Teku J.A., Taylor D.A., Lee J.S. (2025). Thick ZnS Shells on CsPbBr_3_ Quantum Dots by Colloidal-Atomic Layer Deposition for Enhanced Photoluminescence and Stability. JACS Au.

[38] Trinh C.K., Lee H., So M.G., Lee C.L. (2021). Synthesis of Chemically Stable Ultrathin SiO_2_-Coated Core-Shell Perovskite QDs via Modulation of Ligand Binding Energy for All-Solution-Processed Light-Emitting Diodes. ACS Appl. Mater. Interfaces.

[39] Hong Y., Yu C., Je H., Park J.Y., Kim T., Baik H., Tomboc G.M., Kim Y., Ha J.M., Joo J. (2023). Perovskite Nanocrystals Protected by Hermetically Sealing for Highly Bright and Stable Deep-Blue Light-Emitting Diodes. Adv. Sci..

[40] Chen J., Huang X., Xu Z., Chi Y. (2022). Alcohol-Stable Perovskite Nanocrystals and Their In Situ Capsulation with Polystyrene. ACS Appl. Mater. Interfaces.

[41] Dong H., Zhao H., Xuan T., Bai W., Lin T., Cai Y., Xie R.J. (2023). Constructing Perovskite/Polymer Core/Shell Nanocrystals with Simultaneous High Efficiency and Stability for Mini-LED Backlights. ACS Appl. Mater. Interfaces.

[42] Bommireddy P.R., B J.B., Sunku S., C K.B., Suh Y., M C.S., Park S.H. (2024). Highly Stable CsPbBr_3_/PMA Perovskite Nanocrystals for Improved Optical Performance. Heliyon.

[43] Huang S., Bian C., Xu W., Zhang H., Gao S., Wang Y., Wang Y. (2023). Enhanced Stability of CsPbBr_3_ Nanocrystals through Al_2_O_3_ and Polymer Coating. Phys. Chem. Chem. Phys..

[44] Li G., Rivarola F.W.R., Davis N.J.L.K., Bai S., Jellicoe T.C., De La Peña F., Hou S., Ducati C., Gao F., Friend R.H. (2016). Highly Efficient Perovskite Nanocrystal Light-Emitting Diodes Enabled by a Universal Crosslinking Method. Adv. Mater..

[45] Ganswindt P., Tepfenhart I., Singldinger A., Abfalterer A., Spies L., Kostyurina E., Stadler M., Nickel B., Urban A.S. (2025). Locking in Color: Stable RGB Perovskite Nanocrystal Films via UV Cross-Linking. Adv. Opt. Mater..

[46] Ren J., Dong B., Liao Y., Liu L., Zhang C., Zhou X., Liu H., Liu F., Chen L., Wang Y. (2026). Highly Luminescent Photo-Crosslinkable Perovskite Nanocrystals: Advancing Direct Optical Patterning, 3D Painting, and Backlight Displays. Adv. Funct. Mater..

[47] Sun H., Yang Z., Wei M., Sun W., Li X., Ye S., Zhao Y., Tan H., Kynaston E.L., Schon T.B. (2017). Chemically Addressable Perovskite Nanocrystals for Light-Emitting Applications. Adv. Mater..

[48] Han B., Yuan S., Cai B., Song J., Liu W., Zhang F., Fang T., Wei C., Zeng H. (2021). Green Perovskite Light-Emitting Diodes with 200 Hours Stability and 16% Efficiency: Cross-Linking Strategy and Mechanism. Adv. Funct. Mater..

[49] Liu D., Weng K., Lu S., Li F., Abudukeremu H., Zhang L., Yang Y., Hou J., Qiu H., Fu Z. (2022). Direct Optical Patterning of Perovskite Nanocrystals with Ligand Cross-Linkers. Sci. Adv..

[50] Shin S., Kim Y., Gwak N., Jeong I., Lee M., Kang K., Yeon S., Kim S., Kim T.A., Oh N. (2023). Light-Induced Crosslinking of Perovskite Nanocrystals for All-Solution-Processed Electroluminescent Devices. Appl. Surf. Sci..

[51] Xu Y., Guo W., Yao J., Xu L., Wang J., Yang Z., Wang S., Song J. (2025). Multisite Cross-Linked Ligand Suppressing Ion Migration for Efficient and Stable CsPbBr_3_ Perovskite Quantum Dot-Based Light-Emitting Diodes. Angew. Chem. Int. Ed..

[52] Ma J., Yang L., Xue Y., Gao Y., Chen H., Zhang D., Wang Z., Kuang Y., Yu Y., Shao M. (2023). Spatial Cross-Linking Strategy for Realizing Stable and Efficient Perovskite Light-Emitting Diodes toward Microscale Displays. ACS Energy Lett..

[53] Lu W., Wu Y., Li X., Huang J., Ali A., Ren J., Shang Q., Li C., Baryshnikov G.V., Xu B. (2026). Multifunctional Dithiol Crosslinking Additive Enables Highly Efficient and Stable Inkjet-Printed Perovskite Quantum Dot Light-Emitting Diodes. Adv. Mater..

[54] Leo U., Gruber M.A., Henke N.A., Reisner V., Kalkan S.B., Lichtenegger M.F., Nickel B., Urban A.S. (2026). Electron-Beam Cross-Linked Ligands Enable Highly Stable and Freestanding Perovskite Nanocrystal Films. J. Phys. Chem. Lett..

[55] Chen Z., Hoye R.L., Yip H.L., Fiuza-Maneiro N., López-Fernández I., Otero-Martínez C., Polavarapu L., Mondal N., Mirabelli A., Anaya M. (2024). Roadmap on perovskite light-emitting diodes. J. Phys. Photonics.

[56] Thi Ngo L., Lin Y.H., Liu R.S. (2025). Emerging Strategies for Inkjet-Printed Perovskite Quantum Dots in High-Performance LED Displays. Mater. Horiz..

[57] Qing W., Zhang H. (2026). Photocrosslinking Chemistry for Direct Photopatterning of Colloidal Nanocrystals: Toward Pixelated Light Emitting Diodes and Beyond. Chem. Sci..

[58] Gu X., Pu C. (2026). Ligand-Enabled Chemical-Potential Modulation of A-Site Cation Reactivity Stabilizes Tin Halide Perovskite Nanocrystals. Angew. Chem. Int. Ed..

[59] Ali M.U., Sun W.T., Sergeev A.A., Rehman A.U., Wong K.S., Djurišić A.B., Popović J. (2025). Photoluminescence and Stability of Dion–Jacobson Tin-Based Halide Perovskites with Different Spacer Cation Chain Length. Molecules.

[60] Gahlot K., di Mario L., Bosma R., Loi M.A., Protesescu L. (2024). Air-Stable Thin Films of Tin Halide Perovskite Nanocrystals by Polymers and Al_2_O_3_ Encapsulation. Chem. Mater..

